# Sustainable Integration of Nanobiosensors in Biomedical and Civil Engineering: A Comprehensive Review

**DOI:** 10.1021/acsomega.5c00852

**Published:** 2025-06-10

**Authors:** S. Varadharajan, Mrunmayi Gadre, Vidhi Mathur, Kirthanashri S. Vasanthan

**Affiliations:** † Department of Civil Engineering, Manipal Institute of Technology, 76793 Manipal Academy of Higher Education, Manipal, Karnataka 576104, India; ‡ Manipal Centre for Biotherapeutics Research, 76793 Manipal Academy of Higher Education, Manipal, Karnataka 576104, India

## Abstract

Nanobiosensors represent a rapidly advancing class of analytical tools, offering high sensitivity, selectivity, and real-time detection across biomedical, environmental, and structural domains. This review synthesizes foundational quantum phenomena governing sensor response at the nanoscale and explores the integration of pH-responsive polymers to enhance specificity and functional adaptability. Key methodologies in nanobiosensor design and fabrication are examined, encompassing electrochemical, optical, piezoelectric, and field-effect transistor-based systems. Emphasis is placed on diverse applications, including early disease detection, real-time structural integrity assessment, and monitoring of environmental contaminants. Technical challenges such as material stability, signal drift, and manufacturing scalability are critically analyzed alongside emerging advantages such as multiplexing, miniaturization, and low power demand. A sustainable perspective is introduced through discussions on eco-friendly materials, life cycle assessment, and green fabrication processes. By consolidating recent advancements and interdisciplinary approaches, this work provides strategic insights for the development of next-generation nanobiosensors with enhanced performance, environmental compatibility, and translational potential.

## Graphical abstract

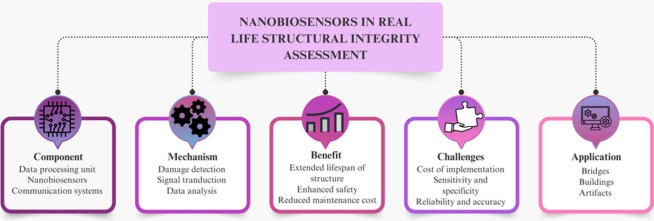

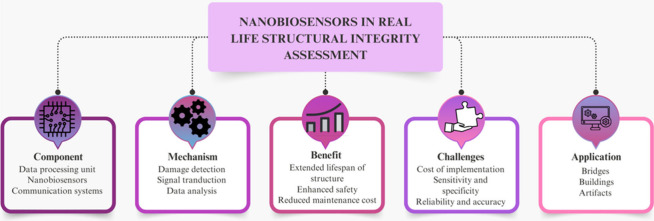

## 1 Introduction

Nanobiosensors have become the new tools that possess exceptional ability in the detection and analysis of different biological and chemical entities, attaining exceptionally high sensitivity and specificity through the convergence of nanotechnology and biological sensing. Nanobiosensors exhibit unprecedented advantage over their traditional counterparts because of their nanoscale,^1,2^ these sensors utilize some unique properties of nanomaterials: High surface-to-volume ratio, quantum effects, and improved reactivity. Thus, nanobiosensors can be used for widespread applications ranging from environmental monitoring^3^ to medical diagnosis,^4^ and health structure monitoring which shows versatility of this technology and its ability to revolutionize existing practices.

Quantum effects and enhanced reactivity play pivotal roles in advancing nanobiosensor performance. The localized surface plasmon resonance (LSPR)^5^ of gold nanoparticles enables ultrasensitive detection through amplified electromagnetic fields. Quantum dots, with their size-dependent emission and sharp spectral profiles, lay the foundation for advanced optical biosensing, while plasmonic nanomaterials such as gold seamlessly extend this functionality by amplifying signal transduction through localized surface plasmon resonance (LSPR), together enabling ultrasensitive, multiplexed detection in nanobiosensor platforms. These properties enable enhanced light–matter interactions at the nanoscale, making Au nanostructures highly effective for both signal transduction and catalytic functionalities in biosensing platforms. Recent developments in advanced nanoarchitecture have significantly expanded their catalytic and optoelectronic performance, laying the groundwork for multifunctional sensor applications.

In the realm of plasmon-enhanced photocatalysis, Au nanostructures have been engineered to achieve remarkable turnover frequencies (TOF) and quantum efficiencies. An et al. reported a TOF of 1533 h^–1^ for hydrogen production using a multisite Au/TiO_2_ photocatalyst system driven by Na^+^-ion-induced self-assembly, illustrating the synergistic role of spatially defined plasmonic hotspots.^6^ Yang et al. further advanced this domain by synthesizing Au nano bipyramid (NBP)/Rh superstructures, achieving 138.2 μmol h^–1^ g^–1^ in nitrogen fixation, showcasing the potential for energy-intensive reactions under mild conditions.^7^ A hybrid AuCu_3_/Cu catalyst fabricated by Li et al. demonstrated a 20-fold enhancement in ammonia generation, underscoring the critical role of bimetallic interfaces in modulating electronic structure and reactivity.^8^

Plasmonic coupling mechanisms have also been leveraged to manipulate photonic transitions. Ha et al. utilized interbond transition engineering in Au/TiO_2_ dumbbell nanostructures to boost oxygen evolution to 0.36 mmol g^–1^ h^–1^,^9^ while Atta et al. demonstrated that spike-shaped Au nanostars grown on TiO_2_ significantly amplified near-infrared (NIR) mediated hydrogen evolution, reaching 2632 μmol g^–1^ within just 20 min.^10^ Similarly, hollow Au–CeO_2_ “nano mushrooms” developed by Li et al. exhibited enhanced infrared absorption and yielded 215.14 μmol g^–1^ h^–1^ of ammonia.^11^ Emulating biological architectures, Chen et al. constructed Au@Nb@HxK_1–*x*_NbO_3_ “plasmonic peapods,″ exploiting strong near-field coupling to broaden the light absorption spectrum.^12^

Further innovations push the plasmonic boundary via heterostructure integration. Lin et al. developed Cu_2_ZnSnS_4_/Au–Au dimer nanocomposites that achieved a 9-fold increase in hydrogen yield (1192 μmol h^–1^ g^–1^), benefiting from multiphoton excitation and interfacial charge transfer.^13^ Incorporation of Au into metal–organic frameworks (MOFs) has also demonstrated full-spectrum photocatalytic activation. Notably, Li et al. engineered an up conversion-Au-MOF-Pt hybrid that efficiently harvested both visible and NIR light for hydrogen evolution,^14^ while Xiao et al. used a Au–Pt–MOF Schottky interface which significantly improved charge carrier dynamics, producing 1743 μmol g^–1^ h^–1^—an enhancement of 170× over baseline MIL-125/Au systems.^15^

Beyond semiconductor-plasmonic coupling, Au nanostructures have facilitated enhanced electrocatalysis and corrosion resistance. Plasmon-assisted activation of molecular catalysts such as cobalt-porphyrins was achieved through LSPR-induced hot carrier injection,^16^ and Zhou et al. demonstrated precise MoS_2_ corrosion modulation via Au nanostructures.^17^ In CO_2_ hydrogenation, Verma et al. introduced black gold-Ni colloidosomes that reached unprecedented productivity (2464 ± 40 mmol gNi^–1^ h^–1^) with 95% selectivity.^18^ This was further refined using DPC-60-derived plasma black gold, which drastically shortened fabrication time while enabling 87% CO conversion without the need for external heating.^19^

These advancements not only reflect the versatility of gold-based nanostructures in catalytic domains but also signal their translability to nanobiosensor platforms. The principles governing plasmon-induced charge separation, spectral tuning, and interfacial engineering provide a foundational framework for designing next-generation biosensors with ultrasensitive detection, spectral multiplexing, and energy-harvesting capabilities. Further, nanobiosensors are finding wide utility in diverse fields. The detailed performance metrics of these plasmonic architectures relevant to biosensing applications are summarized in Table 1.

**Table 1:** Noble Metal Materials^5^

**Plasmonic material**	**Catalyst**	**Photoabsorption region**	**Efficiency**	**Yield/selectivity**	**Year**	**refs.**
Au	Au/TiO_2_	H_2_ from H_2_O	–	2632 μmolH2 gcat ^–1^ in 20 min	2018	^10^
Au	Au/TiO_2_	H_2_ from H_2_O	AQE = 2.3 ± 0.2%	10.1 mmol g^–1^ h^–1^	2018	^20^
Au		H_2_, O_2_ from H_2_O	–	–	2018	^12^
	Au@Nb@Hx K_1–x_NbO_3_					
Au	p-GaN/Au	CO_2_ reduction	–	5 nmol h^–1^	2018	^21^
Au	Au/TiO_2_	–	–	–	2018	^22^
Rh	Rh/TiO_2_	Steam methane reforming	AQE = 7.2%	120 μmol g^1^ min^–1^	2018	^23^
Au	Au/MoS_2_	Hydrogen evolution	–	–	2018	^24^
Au	Au/MIL-125/Pt	H_2_ from H_2_O	–	1743.0 μmol g^–1^ h^–1^	2018	^15^
Pd	Pd/TiO_2_	CO_2_ reduction	–	11.05 μmol h^–1^ g^–1^	2018	^25^
Au	Au/CdS	Deuteration	QE = 1.1%	85%	2019	^26^
Ag	Ag/Ag_3_PO_4_	Reversible ddition–fragmentation chain transfer	–	>99%	2019	^27^
Au	Pt@Au/C_3_N_4_	Au/TiO_2_	–	–	2019	^28^
Au	TiO_2_/Au/CoOx	H_2_, O_2_ from H_2_O	AQE = 0.024%	O2 0.36 mmol g^–1^ h^–1^	2020	^9^
Au	Au/Pt/Ni_2_P	H_2_, O_2_ from H_2_O	–	–	2020	^29^
Au	Au/TiO_2_	H_2_ from H_2_O	–	≈191.2 mmol g^–1^ h^–1^	2021	^30^
Au	Au/TiO_2_	H_2_O_2_ from H_2_O, O_2_	–	–	2021	^17^
Au	Au/Chalcopyrite	H_2_, O_2_ from H_2_O	–	9.1 nmol H_2_ mL^–1^ air	2022	^31^
Au	Au@CeO_2_/Gr	H_2_ from H_2_O	AQE = 38.4%	8.0 μmol mgcat ^–1^ h^–1^	2022	^32^
Au	Au/TiO_2_	H_2_ from H_2_O	–	4.37 mmol g^–1^ h^–1^	2023	^6^
Au	Au/NiO	H_2_ from H_2_O	–	–	2023	^33^
Au	Au/TiO_2_	H_2_ from H_2_O	–	646.0 μmol g^–1^ h^–1^	2023	^34^
Au	Au/CeO_2_	NH_3_ from N_2_	SCCE = 0.1%	215.14 μmol g^–1^ h^–1^	2023	^11^
Au	Cu_2_ZnSnS_4_/Au	H_2_ from H_2_O	–	≈1192 μmol h^–1^ g^–1^	2023	^13^
Ag	Ag/metal–organic	H_2_ from H_2_O	–	–	2023	^35^
	matrix					
Au	Au/CoTPyP	H_2_ from H_2_O	–	–	2023	^16^
Au	Au (black gold-Ni)	CO_2_ reduction	–	2464 ± 40 mmol gNi ^–1^ h^–1^	2023	^18^
Au	Au–SiO_2_	CO oxidation	–	454 mmol g^–1^ h^–1^	2024	^19^

### 1.1 Scope of This Review Paper

This review presents a comprehensive analysis of nanobiosensors, emphasizing their fundamental mechanisms, material innovations, and multidisciplinary applications. Nanobiosensors, by leveraging nanoscale phenomena, offer enhanced sensitivity, selectivity, and real-time detection capabilities, making them indispensable in biomedical diagnostics, environmental monitoring, and structural health evaluation. The review begins by exploring the quantum-level principles that govern sensor performance, including phenomena such as tunneling effects, quantum confinement, and surface energy modulation. It also examines the role of stimuli-responsive polymers—particularly pH-sensitive materials—in enabling smart, adaptive sensing functions suited for dynamic biological and environmental conditions.

Various classes of nanobiosensors are systematically reviewed, including electrochemical-, optical-, piezoelectric-, plasmonic-, and transistor-based platforms. The discussion extends to design strategies and transduction mechanisms, illustrating how biosensors are engineered for specific analyte recognition and signal conversion. Material selection and fabrication techniques are critically assessed, focusing on a broad spectrum of advanced nanomaterials characterized by distinctive electrical, optical, and magnetic properties. Key application areas are highlighted, ranging from point-of-care medical diagnostics and early disease detection to environmental contaminant monitoring and real-time structural integrity assessment in engineering systems. These diverse use cases underscore the technological versatility and societal relevance of nanobiosensor platforms.

In addition, the review introduces a sustainability perspective—addressing eco-friendly material selection, energy-efficient sensor operation, and end-of-life considerations through life cycle assessment. This dimension reflects a growing recognition of the environmental and ethical responsibilities associated with the development of next-generation sensing technologies. The review concludes by identifying prevailing challenges, such as miniaturization limits, long-term operational stability, and translational barriers. Future opportunities are discussed in the context of integrated sensing networks, data-driven optimization, and sustainable design principles. Altogether, this work serves as a comprehensive reference for advancing nanobiosensor research toward robust, multifunctional, and environmentally responsible technologies.

## 2 Fundamentals of Nanobiosensors

### 2.1 Basic Principles and Mechanisms

Nanobiosensors are based on unique properties of nanomaterials like large surface areas,^36–39^ quantum effects, and enhanced reactivity.^39^ These properties empower nanobiosensors to detect and analyze target molecules with very high sensitivity and specificity. In essence, the fundamental principle of nanobiosensors is an interaction between a biological recognition element (for example, enzyme, antibody, or nucleic acid) with a target analyte. This interaction leads to the formation of a measurable signal, which is then transduced into readable output to show the presence or concentration of the target analyte. This foundational mechanism is further exemplified by the signal transduction processes employed in various nanobiosensor platforms. The signal transduction pathway in nanobiosensors can be better illustrated by specific examples. In a fluorescence-based optical biosensor, target DNA hybridization with a fluorophore-quencher-tagged probe causes spatial separation, restoring fluorescence. This optical change is transduced by a photomultiplier tube or CCD detector, which converts the light signal into an electrical output. Signal processing algorithms then quantify the fluorescence intensity, enabling precise analyte concentration measurement. Beyond fluorescence-based systems, other optical transduction mechanisms also offer powerful, label-free detection capabilities.

Optical affinity biosensors based on surface plasmons (SPs) operate by transducing minute refractive index changes at metal-dielectric interfaces into optical signals, enabling the label-free detection of biomolecular interactions in real time. They leverage either propagating surface plasmon polaritons (PSPPs) on planar metal films or localized surface plasmon resonances (LSPRs) in metal nanostructures, including nanorods, nanoholes, and nanoparticles.^40–42^ Recent architectures exploit hybrid plasmonic coupling, Fano-type interferences, and surface lattice resonances (SLRs) to enhance field confinement and spectral resolution.^43^ The electromagnetic field decay profile, characterized by the penetration depth (ranging from <10 nm in LSPR to >1 μm in long-range PSPP), dictates sensitivity to near-surface binding.^44,45^ Resonance is governed by conservation of energy and momentum, where the incident light angle and wavelength determine efficient coupling, observed as distinct spectral dips and peaks in angular, spectral, or intensity modulated configurations. Imaging modalities like SPR microscopy, SPR imaging, and hyperspectral SPR enhance multiplexing and spatial resolution.^46,47^ Excitation typically employs Kretschmann-based total internal reflection using prisms or microscope objectives or grating-mediated diffraction in periodic metallic nanostructures for miniaturization. Nanohole arrays, combined with imaging/spectrometry, offer scalability and high-throughput analysis^48^ while random plasmonic colloids enable naked-eye detection.^49^ Fully integrated SPR biosensors incorporate functional biorecognition layers, microfluidic modules, thermal stabilization, and automated sample preprocessing, delivering high specificity and robustness across diverse biomedical and environmental applications. The literature review pertaining to applications of SPR biosensors are shown in Figures 1–3 and Table 2.

**Figure 1:**
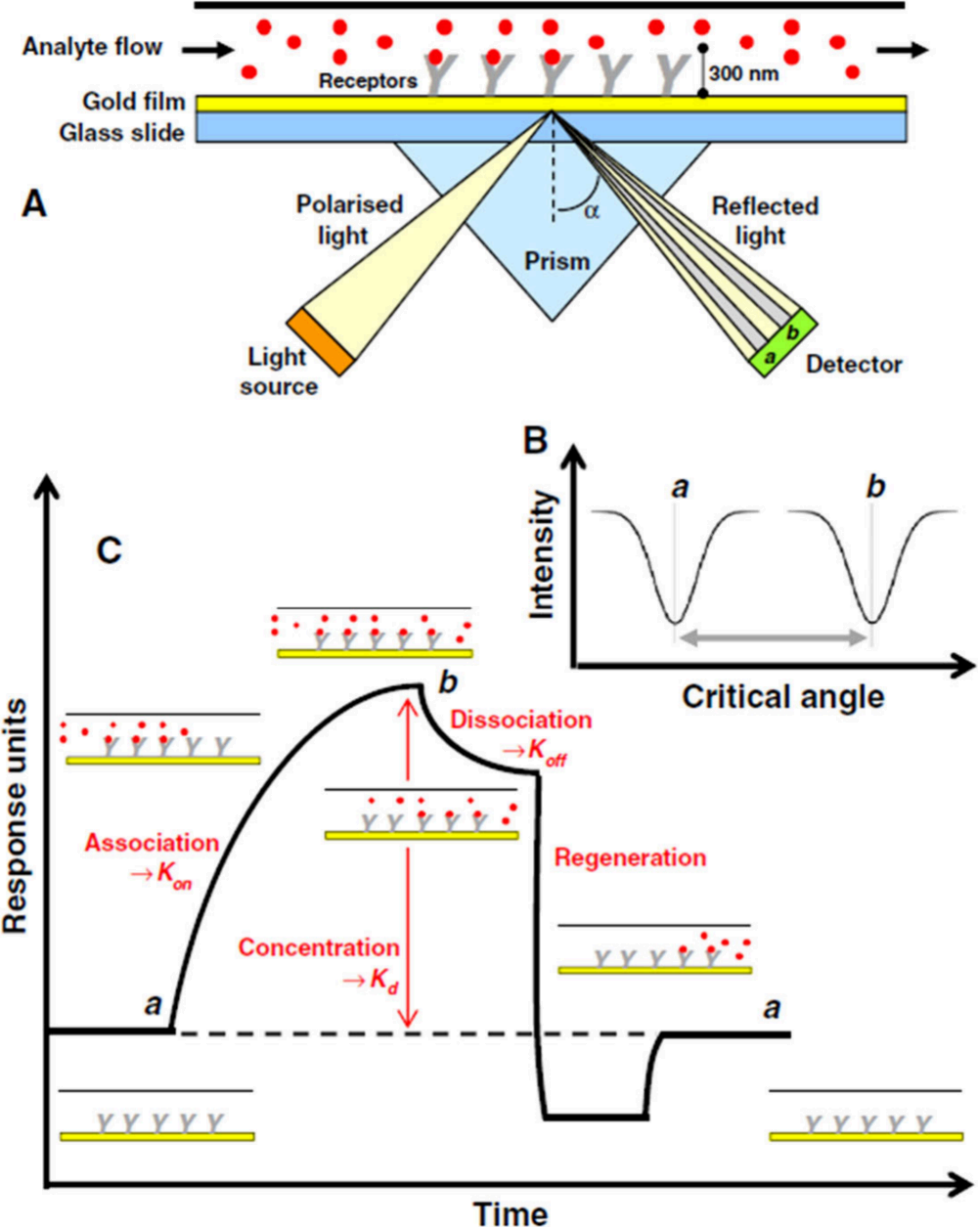
(A) Instrument setup for an SPR experiment. (B) Change in the SPR angle of incident light from angle a to angle b on the binding of an analyte molecule to a bioreceptor molecule. (C) Response of the SPR experiment in the form of a sensogram. Figures (A–C) were reproduced from ref 275 with permission from Elsevier. Copyright 2014, References 276–279 .

**2:**
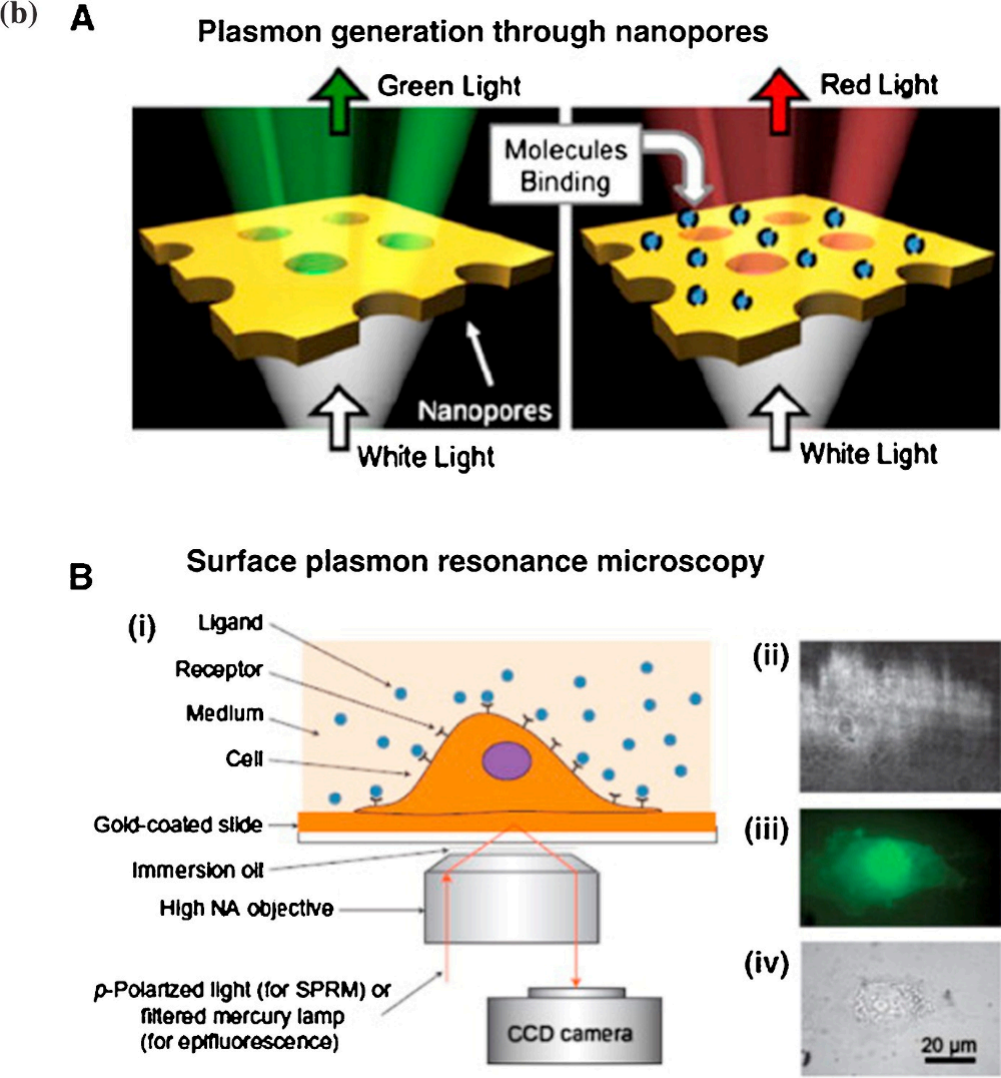
Next-generation SPR instrumentation for measuring membrane protein–ligand binding. (A) Nanopores in a gold film through which there is enhanced transmission of light due to plasmon generation, which undergoes a red-shift on binding of molecules. (B) Surface plasmon resonance microscopy with intact living cells. (i) Schematic illustration of the experimental setup; (ii) SPR image of a cell; (iii) fluorescence image of a cell; (iv) bright-field image of a cell. (Picture A was modified from ref 280 and B was modified from ref 281.) [Were reproduced from ref 275 with permission from Elsevier. Copyright 2014].)

**Figure 3:**
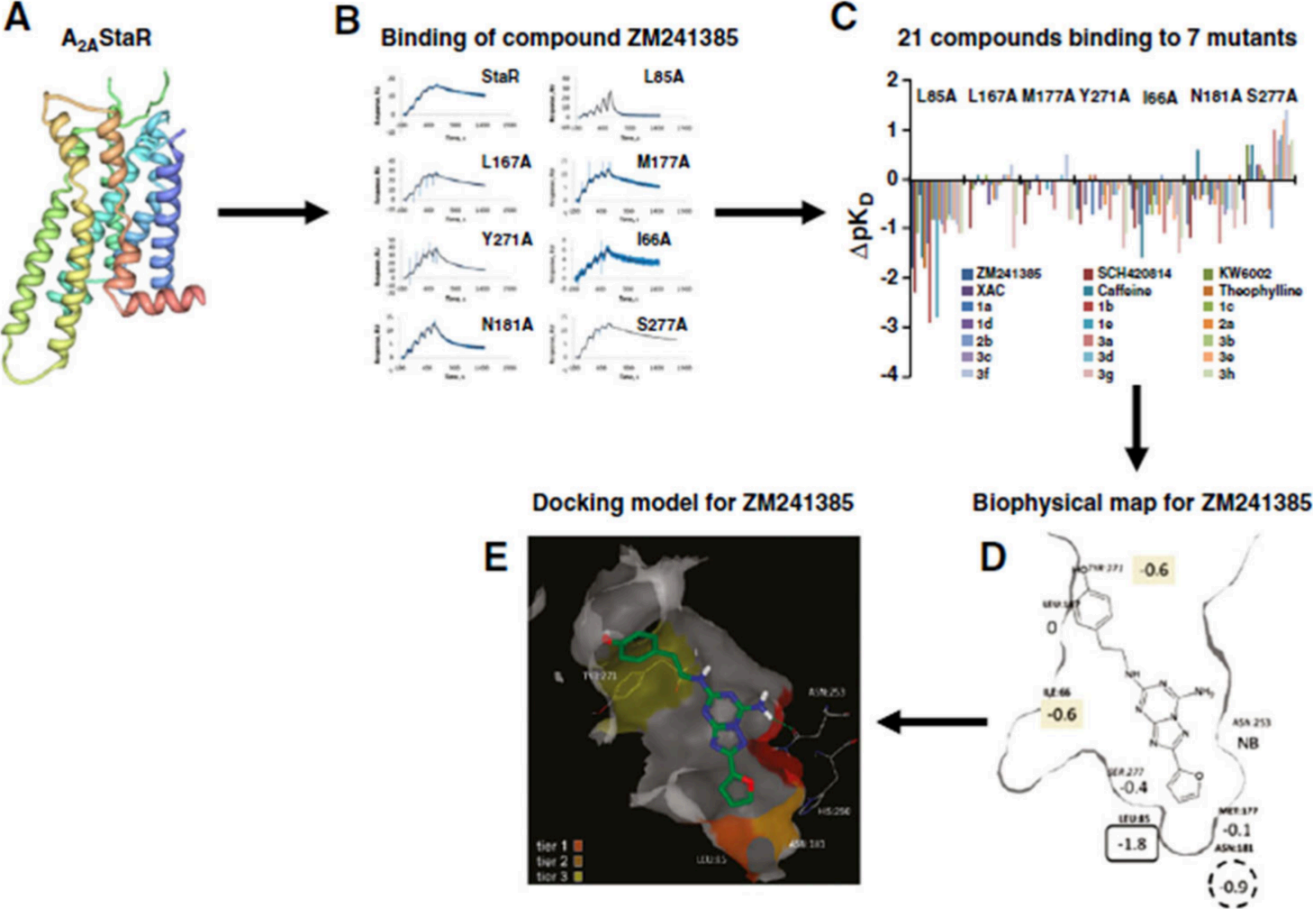
Biophysical mapping of the adenosine A2A receptor using SPR. (A) The procedure starts with a stabilized receptor (StaR) minimally engineered for thermostability. (B) Sensorgrams for binding of compound ZM241385 to StaR and mutant forms of A2A. Following introduction of further single mutations at positions proposed to be in the ligandbinding site, SPR measurements of ligand binding are performed on the StaR and mutant receptors. (C) Matrix of SPR responses (as a log difference compared with unalteredStaR background) for 21 compounds binding to 7 different mutants of A2A. (D) Biophysical representation from the SPR data for compound ZM241385 based on a homology model of A2A. Each of the shown residues was mutated to alanine, and the log difference value for binding compound ZM241385 is shown. Key: residues in bold font = in front of the plane, italics = behind the plane, normal font = in the plane of the ligand, NB = nonbinding, black oval = largest effect, dotted circle = second largest effect, shaded box = third largest effects. E. Docked structure of compound ZM241385 in a homology model of A2A. Asn 253 is colored red as mutation of this residue prevents binding of ligands.The first, second and third tier effects of mutations are colored according to the key and relate to the residues indicated in D from the BPM data. (Picture A was produced using the PDB file (PDB ID: 3PWH) and PDB Protein Workshop 3.9 from ref 282; B was modified from ref 282; C was constructed from data given in ref 283; D and E were reproduced from ref 283.) [Were reproduced from ref 275 with permission from Elsevier. Copyright 2014].

**Table 2:** (a) Formulas Related to SPR and Protein Binding Kinetics [Equations Reported in Ref 40] and (b) Selected Examples of Applications of SPR Biosensors Developed for the Detection of Chemical and Biological Species^50^

**(a)**	
**Formula**	**Parameter Descriptions**
(∂n_e_ f /∂n_d)_B = n_e_ f ^3^/n_d^3^ > 1	n_e_ f : Effective refractive index of surface plasmon; n_d: Refractive index of dielectric medium; This equation shows that the effective index change is more sensitive than the bulk medium index change.
(∂n_e_ f /∂n_d)_S = 2 (∂n_e_ f /∂n_d)_B × (h/L_pd)	h: Thickness of the sensitive layer; L_pd: Penetration depth of the surface plasmon; This equation expresses surface sensitivity based on bulk sensitivity and effective field penetration.
σ_RI = K × (1/√N) × (σ_th/d) × (w/S_RI)	σ_RI: Refractive index noise; N: Number of intensities; σ_th: Intensity noise at threshold; d: Dip intensity difference; w: Width of dip; S_RI: Bulk refractive index sensitivity; K: Noise factor.
σ_Γ = (σ_SO × h)/(S_h × (∂n/∂c)_vol)	σ_Γ: Minimum resolvable surface concentration; σ_SO: Sensor output noise; h: Layer thickness; S_h: Sensitivity of sensor output to RI changes; (∂n/∂c)_vol: RI increment with concentration.
σ_Γ = (σ_RI × L_pd)/(2 × (∂n/∂c)_vol)	σ_RI: Refractive index resolution; L_pd: Plasmon penetration depth; (∂n/∂c)_vol: Volume RI increment; This shows link between RI resolution and mass detection limit.
(2π/λ) × n_p × sin(θ) = Re{β_SP}	λ: Wavelength of light; n_p: Refractive index of prism; θ: Incident angle; β_SP: Propagation constant of the surface plasmon. This is the matching condition for SPR excitation.
dR(t)/dt = k_a C (R_max – R(t)) – k_d R(t)	Real-time association kinetics:
	R(t): Response at time t (in resonance units, RU); k_a: Association rate constant (M^–1^s^–1^); k_d: Dissociation rate constant (s^–1^); C: Analyte concentration (M); R_max: Maximum binding capacity (RU)
R(t) = R_0 e^(‑k_dt)^	Dissociation kinetics:
	R(t): Response at time t during dissociation; R_0: Initial response at start of dissociation; k_d: Dissociation rate constant (s^–1^); t: Time (s)
R_eq = (R_max × C)/(K_D + C)	Equilibrium binding response:
	R_eq: Response at equilibrium (RU); R_max: Maximum binding response (RU); C: Analyte concentration (M); K_D: Equilibrium dissociation constant (M)

**(b)**					
**Pathology**	**Biomarker**	**Detection format (detection time)**	**Performance of the biosensor**	**Other clinically relevant details**	**ref.**
**RNA**					
Multiple sclerosis	miR-422, miR-223, miR-126, miR-23a	SA: Ab-AuNPs (100 min)	LOD: 0.55 (miR-422), 0.88 (miR-223), 1.19 (miR-126), and 1.79 pM (miR-23a)	CS: C = 3 (total RNA isolates from blood serum)	^51^
Diabetic nephropathy	miR-21, miR-192	SDA + SA: DNA-AuNPs (150 min)	LOD: 0.15 (miR-21), and 0.22 pM (miR-192)	RT: 103–110% (10% fetal bovine blood serum)	^52^
Nonsmall cell lung cancer	miR-21, miR-378, miR-139, miR-200	SA: DNA-AgNCs + DNA-AuNPs (240 min)	MC: 2 fM to 20 nM	CS: *P* = 5, C = 5 (RNA isolated from exosomes from blood plasma); elevated in P	^53^
Esophageal squamous cell carcinoma	miR-21, miR-155	SA: DNA-Fe3O4@AuNPs (120 min)	LOD: 502 (miR-21), and 483 fM (miR-155) (10% blood serum)	CC (10% blood serum)	^54^
Myelodysplastic syndromes	miR-125b, miR-16	Release of specifically captured DNA-AuNPs (120 min)	LOD: 350 (miR-125b), and 550 aM (miR-16) (10% blood plasma)	CC (10% blood plasma)	^55^
Glioma	miR-182	DNA walking system + SA: streptavidin	LOD: 620 aM	CS: *P* = 3, C = 3 (20% blood serum); elevated in P	^56^
**DNA**					
Thalassemia	β-globin gene mutations	PCR + DD (15 min)	–	CS: *P* = 52, C = 19 (DNA isolates from blood or salivary swabs); P and C differentiation	^57^
Pneumocystis pneumonia	Pneumocystis jirovecii DNA	DD (15 min)	LOD: 2.1 nM	CS: *P* = 4, C = 8 (DNA isolated from sputum or bronchoalveolar lavage); P and C differentiation	^58^
Colorectal cancer	RAS ctDNA	SA: DNA-AuNPs (80 min)	LOD: 2 ng/mL (10% blood plasma)	CS: *P* = 4, C = 4 (10% blood plasma); P and C differentiation	^59^
Colorectal cancer	KRAS ctDNA	SA: DNA-AuNPs (120 min)	LOD: 1.45 ng/mL (10% blood plasma)	CS: *P* = 1, C = 1 (10% blood plasma); elevated in P	^60^
Pancreatic cancer	KRAS ctDNA	DD (5 min)	LOD: 25 pg/mL (100% blood serum)		^61^
**Protiens**					
Colorectal cancer	hnRNP A1	SA: Ab (70 min)	LOD: 0.22 nM	RT: ∼15% (blood plasma)	^62^
Myeloma	IL-2, sIL-2Rα	DD (1 min)	LOD: 20 nM	CS: *P* = 2, C = 2 (0.1% blood serum); elevated in P	^63^
Bladder cancer	NMP22, CPH, HA	SA: Ab-AuNPs (120 min)	MC: 0.001–1000 ng/mL	RT: 80–120% (10% fetal bovine serum)	^64^
Breast cancer	CA 15–3, CA 125, CEA, ErbB2	SA: Ab (90 min)	LOD: 1.85 (CA 15–3), 1021 u/mL (CA 125), and 76.19 (CEA), 31.9 ng/mL (ErbB2) (blood serum)	RT: 77–126% (100% blood serum)	^65^
Myelodysplastic syndromes	misfolded proteins	DD (1 min)	–	CS: *P* = 45, C = 16 (10% blood serum); elevated in P	^66^
Small cell lung cancer	NSE	DD (5 min)	LOD: 15.6 nM (1% blood serum)	CC (1% blood serum)	^67^
Acute myocardial infarction	TnT	DD (2 min)	LOD: 14.8 nM	–	^68^
Heart failure	BNP	SA: Ab-MNPs + DNA-AuNPs (10 min)	MC: 0.1 pg/mL - 10 ng/mL	RT: 92–114% (100% blood serum)	^69^
Alzheimer’s disease	Aβ1–40, Aβ1–42, τ	DD (60 min)	MC: 10 fM - 100 nM (blood plasma)	CC: blood plasma	^70^
Alzheimer’s disease	τ	DD (60 min)	MC: 10 fM - 100 nM (blood plasma)	CS: *P* = 5, C = 1 (blood plasma); elevated in P	^71^
Alzheimer’s disease	Aβ1–42	DD (5 min)	MC: 2 fg/mL - 400 ng/mL	–	^72^
Multiple sclerosis	Ab(GA1, GM1, GT1b)	DD (30 min)	LOD: 17.6 (GA1), 11.3 (GM1), and 8.2 ng/mL (GT1b) (GT1b: 2.34 ng/mL in 10% blood serum)	cross-reactivity (10% blood serum)	^73^
Neurodevelopmental disorders	SCG2	SA: HRP-streptavidin/Ab (150 min)	LOD: 0.016 ng/mL (10% blood serum)	CS: *P* = 8, C = 5 (10% blood serum); elevated in P	^74^
Acute kidney injury	NGAL, IL-18, RBP	SA: Ab-MNPs (45 min)	LOD: 0.19 (NGAL), 0.51 (IL-18) and 0.7 ng/mL (RBP) (50% urine)	CS: *P* = 2, C = 1 (50% urine); elevated in P	^75^
Nephrotic syndrome	HSA, kappa, lambda, B2M	DD (5 min)	LOC: 0.36 (HSA), 0.05 (kappa), 0.1 (lambda), and 0.04 μg/mL (B2M)	CS: *P* = 7, C = 5 (0.1–10% urine); elevated in P	^76^
Peanut allergy	allergen-specific IgE	SA: Ab-MNPs (10 min)	LOD: 5 pg/mL (diluted calf serum)	CS: *P* = 19, C = 13 (0.01–0.1% blood serum); elevated in P	^77^
Celiac disease	GIP	Indirect competitive assay (10 min)	LOD: 1.6 ng/mL (100% urine)	RT (*n* = 21): 96–101% (100% urine)	^78^
Dengue fever	NS1	DD (10 min)	MC: 0.08–800 nM	CS: *P* = 16, C = 10 (1% blood plasma); elevated in P	^79^
COVID-19	S1 spike protein	DD (10 min)	LOD: 0.26 nM	RT: 95 ± 18% (1% saliva)	^80^
COVID-19	S proteins of Alpha, Beta, and Gamma	DD (20 min)	MC: 100 fM - 100 nM (blood serum)	CC (blood serum)	^81^
Tuberculosis	HspX	DD (20 min)	LOD: 0.6 ng/mL (50% sputum)	CS: *P* = 12, C = 22 (50% sputum); elevated in P	^82^
COVID-19	Ab (SARSCoV-2 S protein)	DD (20 min)	LOD: 0.1 ng/mL	RT: 80–95% (0.1% blood plasma)	^83^
COVID-19	Ab (SARSCoV-2 S protein)	SA: Ab (10 min)	LOD: 1.35 ng/mL	–	^84^
COVID-19	Ab (SARS CoV-2 RBD and S protein)	DD (30 min)	LOD: 21.1 (Ab(RBD)), and 86 ng/mL (Ab(N)) (10% blood serum)	CS: *P* = 100, C = 20 (10% blood serum); elevated in P	^85^
COVID-19	IgG, IgM, IgA Ab (SARS-CoV-2 RBD protein)	SA: Ab (30 min)	–	CS: *P* = 53, C = 49 (10% blood serum); elevated in P	^86^
COVID-19	Ab (SARS-CoV-2 S protein)	DD (2 min)	LOC: 10 nM	CS: *P* = 13 (vaccinated donors), C = 3 (10% blood plasma); elevated in P	^87^
COVID-19	IL-6	DD (30 min)	LOD: 4.6 pg/mL (blood serum)	CS: *P* = 7 (with treatment), C = 9 (without treatment) (blood serum); elevated in P	^88^
Hepatitis A	Ab (HAV)	DD (10 min)	LOD: 20 pM	CS: *P* = 2, C = 5 (0.1% blood serum); elevated in P	^89^
Breast and lung cancer	TGF-β	DD (30 min)	MC: 10 pg/mL to 100 μg/mL	–	^90^
Lymphoma	IL-2	DD (50 min)	LOD: 45.7 pM	–	^91^
Tuberculosis	IFN-γ	DD (5 min)	LOD: 50 pM	–	^92^
**Exosomes**					
Breast cancer	HER2-positive exosomes	SA: tryramide-AuNPs (60 min)	LOC: 10^4^ exosomes/mL	CS: *P* = 8, C = 8 (exosomes isolated from blood serum); elevated in P	^93^
Alzheimer’s disease	CNS-derived exosomes	DD (20 min)	–	CS: *P* = 10, C = 10 (exosomes isolated from blood serum); elevated in P	^94^
Hepatic cancer	SMMC-7721-derived exosomes	SA: DNA-AuNPs + HAuCl_4_ (100 min)	LOD: 5.6 × 10^8^ exosomes/mL	RT: 95–104% (50% blood plasma)	^95^
Breast cancer	Commercial MCF derived exosomes	DD 40 Min	Detection of individual exosome binding	–	^96^
**Viruses**					
COVID-19	CoV NL63 coronavirus	DD (2 s)	MC: 625–10^4^ PFU/mL (90% saliva)	CC (90% saliva)	^97^
Avian influenza	Influenza A H7N9 virus	DD (5 min)	MC: 10^3^–10^5^ copies/mL (10% nasal mucosa solution)	CC (10% nasal mucosa solution)	^98^
**Bacteria**					
Hemolytic uremic syndrome	*E. coli*	DD (2 min)	LOC: 10^3^–10^6^ CFU/mL	Serotyping (correct identification of 186 from 188 isolates)	^99^
Urinary tract infection	*E. coli*	DD (15 min)	MC: 10^3^–10^9^ CFU/mL (urine)	CC (urine)	^100^
**CTFs**					
Acute myeloid leukemia	AML cancer stem cells	DD (5 min)	LOC: 1 × 10^5^ cells/mL	CS: *P* = 7, C = 1 (isolates from blood or bone marrow); elevated in P	^101^
Breast cancer	MCF-7 breast cancer cells	DD (15 min)	LOC: 1 × 10^5^ cells/mL (whole mouse blood)	–	^102^

### 2.2 pH-Responsive Polymers: Smart Materials Tuned by Environmental Stimuli

Stimulus-reactive polymers are intelligent macromolecules that respond to environmental triggers such as pH, temperature, light, and voltage by altering their structure and properties.^103,104^ Among them, pH-responsive polymers—containing ionizable groups like–COOH,–SO_3_H,–PO_4_H, pyridine, and amines—undergo reversible protonation/deprotonation, modulating solubility, conformation, and self-assembly. These polyelectrolytes are categorized as anionic e.g., PAA,^105^ PMAA,^106^ sulfonic,^107^ and phosphate-based,^108^ or cationic e.g., methyl acrylates,^109^ poly­(2-vinylpyridine),^110^ polypyridine,^111^ polylysine,^112^ and dendritic amines.^113^ Anionic polymers expand in basic media via ionization,^114^ while cationic variants swell under acidic conditions. Such transitions lead to flocculation, micellization, hydrogel swelling, or precipitation and enable advanced applications in biosensing, drug/gene delivery, chromatography, and smart membranes. Dual pH-thermal responsive systems^109^ enhance biomedical control, while natural and synthetic polymers are both explored for target-specific release. With growing interest, this Perspective aims to classify pH-responsive systems, outline their molecular mechanisms, and highlight innovations across applied domains.

The synergistic mechanism between self-healing materials and nanosensors involves a closed-loop system, wherein real-time damage detection directly initiates site-specific repair. Embedded nanosensors—such as piezoresistive, mechanochromic, or thermo/electro-responsive devices—detect microcracks, delamination, or thermal stress with high spatial precision. Upon reaching a critical threshold, the sensor transduces these physical stimuli into an activation signal (e.g., localized Joule heating, electric field generation, or catalytic pH shift). This signal triggers the release of the restorative agent by rupturing microcapsules, opening microvalves in vascular channels, or altering the permeability of stimulus-responsive membranes. The result is an on-demand, autonomous release of healing compounds, ensuring damage mitigation is both localized and temporally aligned.^103^ This synergy enhances the material’s durability, responsiveness, and functional longevity under dynamic loading conditions (Tables 3 and 4).

**Table 3:** pH Response to Microcapsules and Their Applications^103^

**Application**	**Experimental methods**	**Experimental operation**	**Reference**
Drug release, antibacterial	Pickering emulsion interfacial polymerization	When pH-stimulated response microcapsules were added to CNF membranes, the cumulative release of cinnamaldehyde increased, the pH value decreased, and the microcapsule addition increased	^115^
Improve plant essential oil stability	Polylactic acid microcapsule method	TTO was coated with PLA modified with octenoate chitosan (OSA-CS) as shell material to form microcapsules with long-term antibacterial and pH response	^116^
Wastewater Treatment	Microsuspension iodine transfer polymerization	Photocatalyst poly(methyl methacrylate–methyl acrylate–divinylbenzene) (P (MMA-MA-DVB)) microcapsules encapsulated with bismuth vanadate (BiVO_4_) and magnetite (Fe_3_O_4_) NPs	^117^
Citral controlled release	The pH response controls the release of citral	The tannin–FeIII complex (citral @TA–FeIII) builds citral microcapsules,	^118^
Control the release of pesticide live substances Slow release antibacterial	Photothermal controlled release method Compound emulsion method	Encapsulation of CS and tannin–iron complex photothermal layer in AVM Enzyme-catalyzed pH double-reactive poly(lactate coglycolic acid) @chitosan@capsaicin (CAP@CS@PLGA) double-core–shell microcapsules	^119^,^120^

**Table 4:** pH-Responsive Hydrogels and Their Applications^103^

**Application**	**pH-response results**	**Experimental method**	**Reference**
Adjust the properties of the microgel	pH > 9.5 APMH aggregates and cannot be synthesized	Apply varying amounts of the APMH is incorporated into poly(*N*-isopropylacrylamide) (PNIPAM) as a cationic copolymer	^121^
Medical dressing, drug release	At a pH of 5–8, the dissolution rate of hydrogels increases from 200g/g to 1600g/g within 48 h	BC/PAA pH-responsive hydrogel was prepared using BC as dressing base and acrylic acid (AA) as monomer	^122^
Cross-linked polymer hydrogels	Polymer hydrogels with 10% cross-linking agent added at pH 9–10 have a maximum swelling rate of 680% in 4–5 days	The network polymer with high swelling rate was synthesized by cross-linking of PAA with different contents of N and *N*-methylene bis(acrylamide)	^123^
Intelligent regulation of temporary blocking agent	pH 4, rapidly broken within 1 h	By reacting the primary amino group of the polyvinyl imide structural unit with the benzaldehyde group of the modified polyethylene glycol, a dynamic imine bond is formed to achieve the gel-fusion transition. A kind of smart hydrogel with pH response and reversible gel fusion transition was prepared	^124^

The principles, applications and types of nanobiosensors have been illustrated in Figure 4 and Tables 5 and 6.

**Figure 4:**
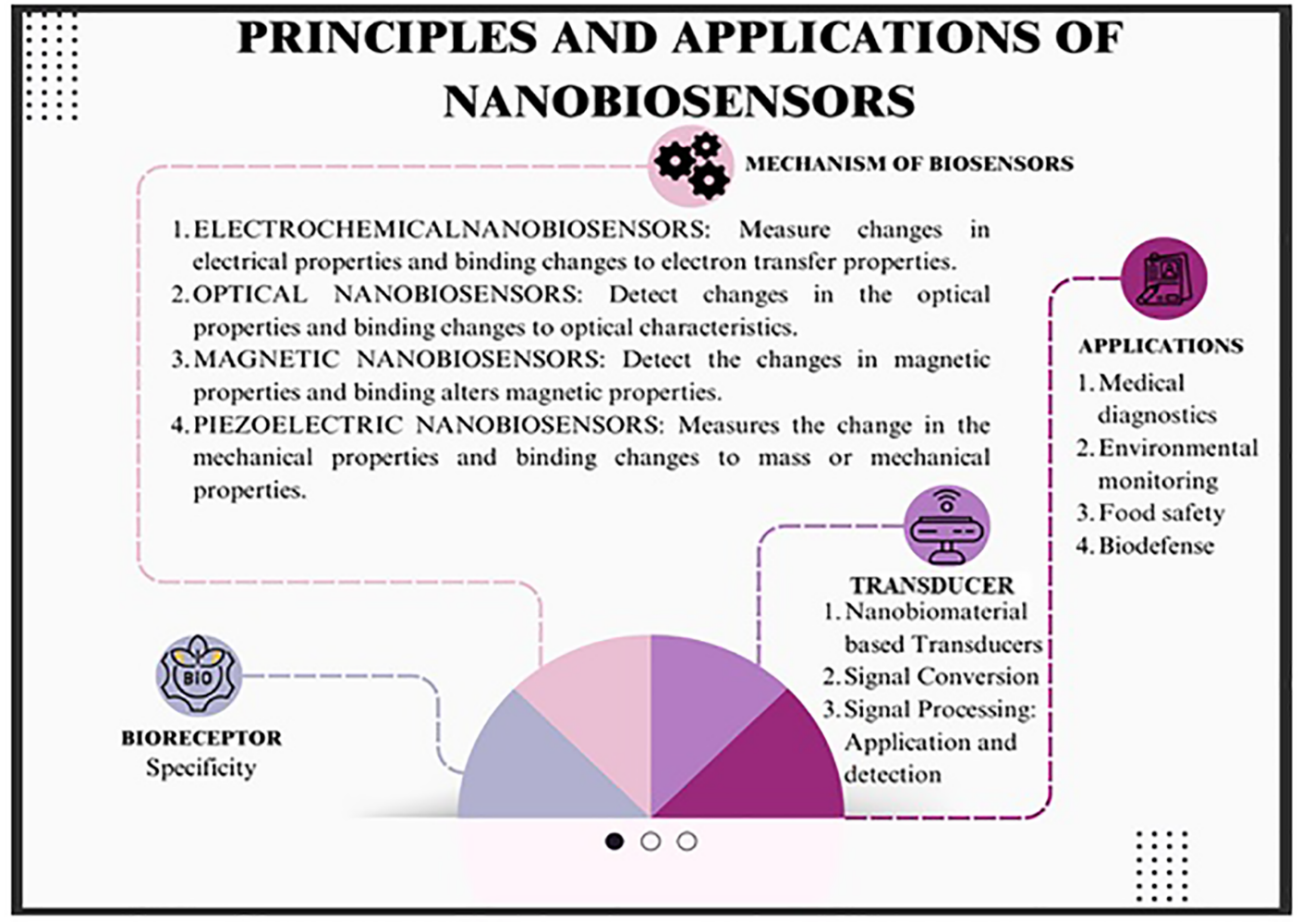
Principles and applications of nanobiosensors.

**Table 5:** Principles, Features, Mechanisms, and Applications of Various Nanobiosensors

**Type of Nanobiosensor**	**Key Features**	**Basic Principle**	**Sensing Mechanism**	**Common Applications**
Electrochemical	High sensitivity, rapid response, miniaturization potential, low detection limits	Monitors electrochemical changes upon biorecognition events	Electroactive species cause modulation in current, potential, or impedance at electrode interfaces using techniques like voltammetry or amperometry	Blood glucose monitoring, detection of neurotransmitters, heavy metals, environmental toxins
Optical	Label-free detection, high spatial and temporal resolution, multiplexing capability	Detects changes in optical properties due to analyte interaction	Analyte binding causes variations in fluorescence intensity, absorbance, luminescence, or refractive index measured via SPR or evanescent wave sensors	DNA hybridization, pathogen detection, optical immunoassays
Piezoelectric	Real-time, label-free mass detection, high precision	Utilizes piezoelectric crystals to detect mass or mechanical perturbations	Binding of analyte alters oscillation frequency or wave propagation in quartz crystal microbalance (QCM) or surface acoustic wave (SAW) devices	Bacterial detection, antigen–antibody interactions, drug discovery
Thermal	Low reagent consumption, sensitive to metabolic heat changes	Measures thermal variations resulting from biochemical reactions	Exothermic/endothermic analyte reactions change temperature, captured by thermistors, calorimetric or thermoelectric transducers	Enzymatic activity monitoring, calorimetric biosensors, clinical metabolism analysis
Magnetic	Nonoptical interference, compatible with turbid or colored samples, high-throughput capability	Detects magnetic field changes due to biorecognition	Functionalized magnetic nanoparticles cluster or shift magnetic field upon target binding, detected by GMR sensors or magnetic resonance	Cancer marker screening, molecular imaging, lab-on-chip diagnostics
Mechanical	Ultrasensitive nanomechanical transduction, label-free, compatible with microfluidics	Relies on mechanical property changes of micro/nano devices	Analyte binding induces stress/strain on cantilevers or nanowires causing deflection or resonance frequency shift	Single molecule detection, real-time protein interaction studies, nanoelectromechanical systems (NEMS)
Plasmonic	High refractive index sensitivity, real-time kinetics, surface-selective	SPR monitors refractive index changes at a metal-dielectric interface	Analyte binding changes local refractive index, modulating resonance angle/wavelength of surface plasmons in metallic nanostructures (e.g., Au, Ag)	Protein interaction studies, biospecific diagnostics, virus detection
Impedimetric	Simple fabrication, scalable integration, suitable for disposable biosensors	Measures electrical impedance variations due to molecular recognition	Analyte alters electrical double layer or charge transfer resistance, recorded via impedance spectroscopy	Detection of pathogens, environmental contaminants, label-free biosensing
Field Effect Transistor (FET)	High sensitivity, label-free, suitable for flexible and wearable sensors	Senses variations in surface potential modulating channel conductance	Target binding modulates gate voltage affecting source-drain current in semiconductor channel (e.g., graphene FET, silicon nanowire FET)	Viral load estimation, hormone detection, cellular microenvironment analysis

**Table 6:** Signal Transduction Mechanism

**Signal Transduction Mechanism**	**Detection Method**	**Application**
Electrochemical	Changes in current, potential, or impedance	Biosensing in medical diagnostics, environmental monitoring
Optical	Changes in fluorescence, absorbance, or SPR signals	Biomolecular interactions, DNA/RNA detection
Piezoelectric	Changes in frequency or wave propagation	Mass-sensitive detection in gas sensors, biosensors
Thermal	Changes in temperature	Thermal analysis in biochemical reactions
Magnetic	Changes in magnetic field	Detection of biomolecules using magnetic nanoparticles
Mechanical	Deflection or frequency change in microcantilevers or nanowires	Mechanical stress or strain detection in biosensing
Plasmonic	Changes in refractive index near sensor surface	Label-free detection of biomolecular interactions
Impedimetric	Changes in impedance	Electrochemical impedance spectroscopy for biosensing
Field Effect Transistor (FET)	Modulation of current through the transistor	High sensitivity detection in biochemical sensors

### 2.3 Design and Fabrication aspect of Nanobiosensors

The design and fabrication of nanobiosensors involve several key considerations: Nanometric dimensions in materials can be created using the top-down approach,^125^ which involves reducing bulk materials to nanoscale sizes through methods like ball milling, lithography, or etching, and the bottom-up approach, which builds nanostructures atom by atom or molecule by molecule using techniques such as chemical vapor deposition, sol–gel processes, or self-assembly.^126^ Additionally, the template-based approach employs preformed molds or scaffolds, such as anodized aluminum oxide, to grow or deposit nanostructures within the templates, enabling precise control over their dimensions. This method is particularly advantageous when working with advanced nanomaterials selected for performance enhancement, such as gold nanoparticles,^127^ carbon nanotubes,^128^ or quantum dots,^129^ as it facilitates their structured integration into devices with high spatial precision and functional efficiency. These materials are chosen based on, a) Compatibility with the biological recognition element and b) Signal transduction properties. Thereafter, The biological recognition element is impregnated onto the nanomaterial surface^130^ to ensure specific binding with a target analyte^125^ using chemical modification or a bioconjugation technique. Finally, the prepared functionalized nanomaterial will then be implemented into an appropriate platform, like a microfluidic device or a wearable sensor, to constitute a nanobiosensor system.

### 2.4 Balancing Innovation and Limitations in Nanobiosensor Applications

Nanobiosensors offer high sensitivity,^131,132^ specificity,^130^ and quick response time.^133^ In addition, they have nanoscale dimensions that enable the realization of portable, miniaturized devices whose range of applications includes point-of-care diagnostics and environmental monitoring. However, nanobiosensors are subjected to some limitations like potential for biofouling, stability issues, extensive fabrication, and integration issues. These limitations are subjects of recent research aimed to improve nanobiosensors in terms of the required robustness, reliability, and cost-effectiveness. The signal transduction mechanism is mentioned in Figure 5 and Table 6. Figure 6 shows Pros and Cons of different types of nanobiosensors.

**Figure 5:**
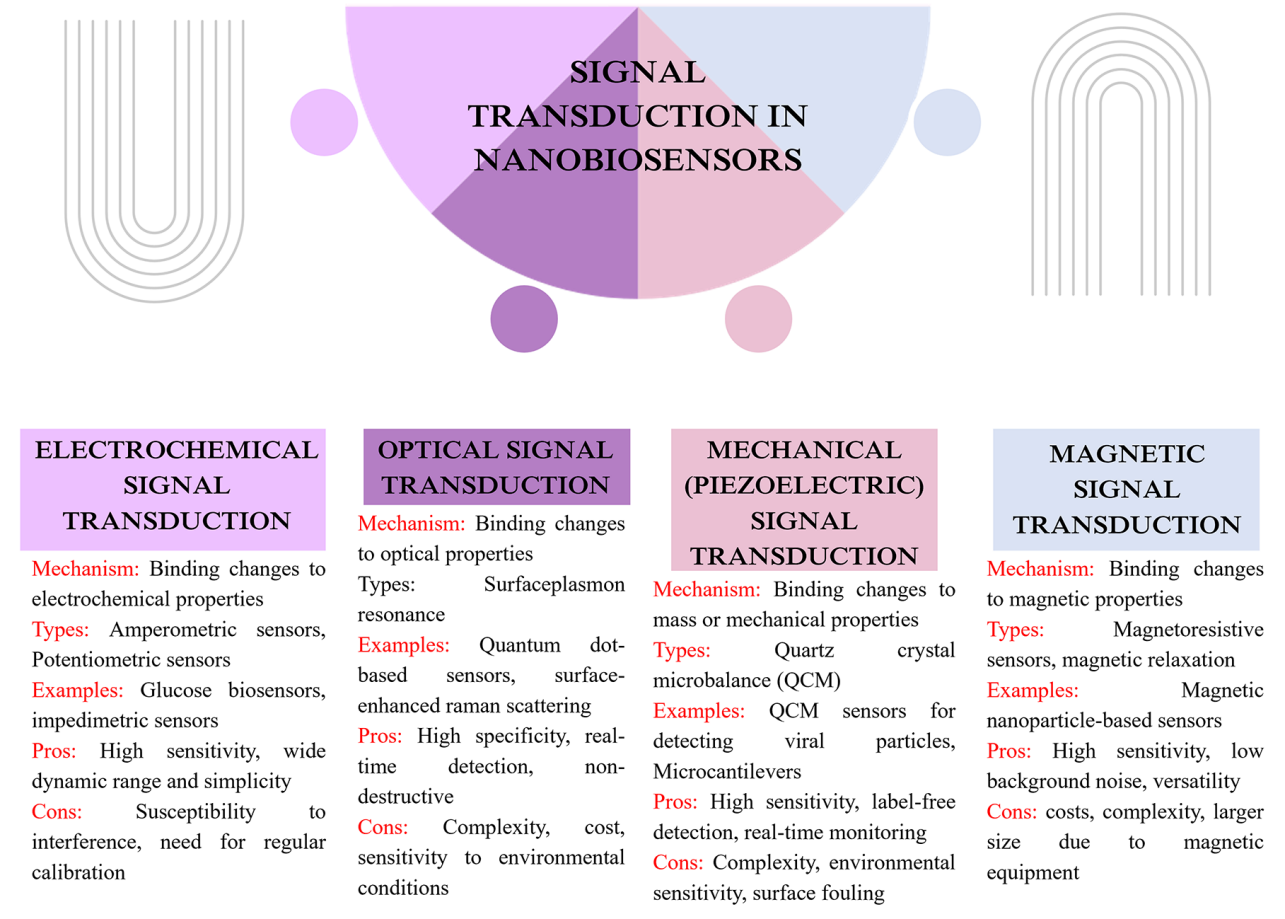
Signal transduction in nanobiosensors.

**Figure 6:**
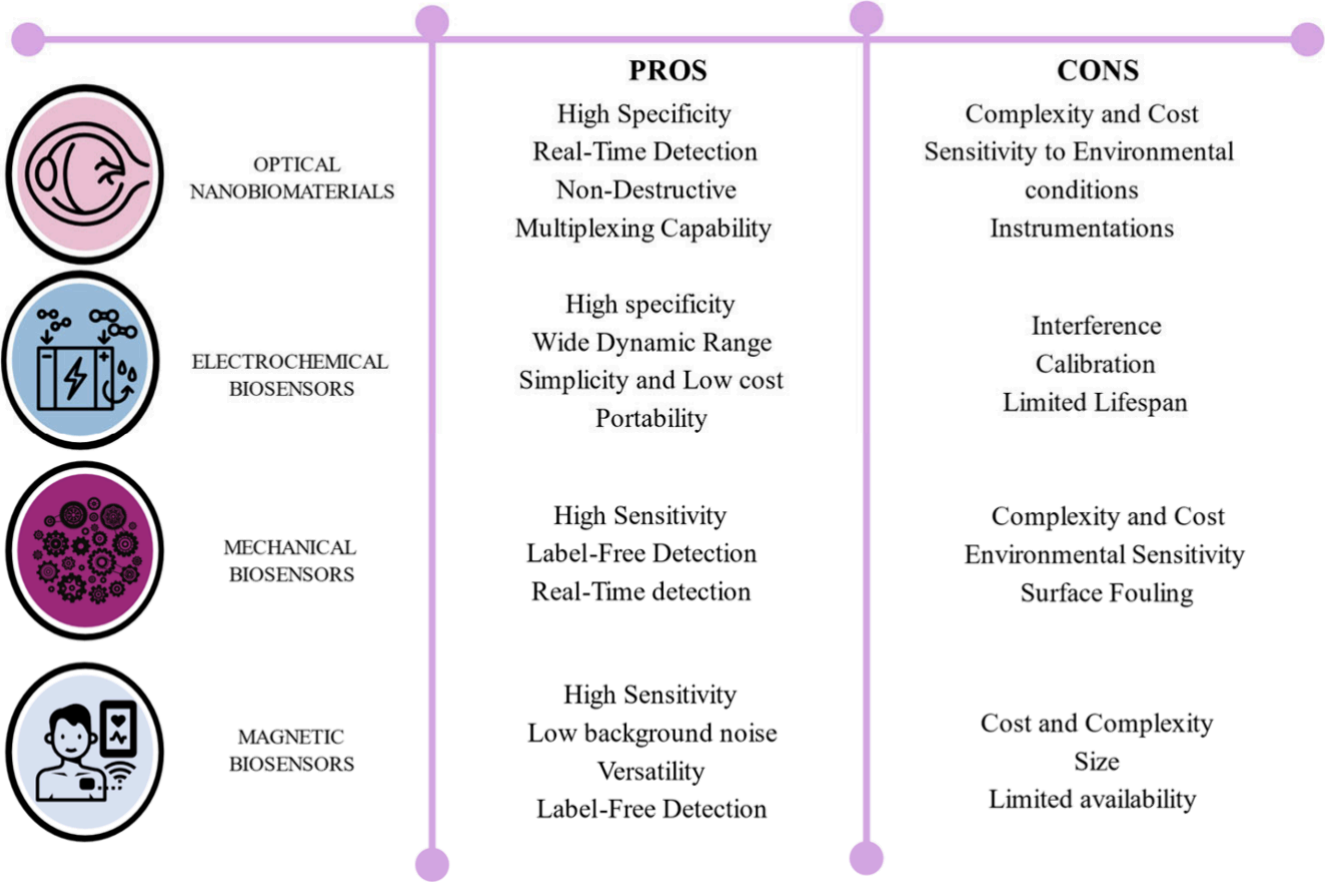
Pros and cons of different types of biosensors.

### 2.5 Types of Nanobiosensors

Nanobiosensors can be classified based on their transduction mechanisms, type of nanomaterials used, and application purposes. The primary forms of nanobiosensors are as follows:

***Optical nanobiosensors*** are based on changes in the optical properties of fluorescence, luminescence, absorbance, or surface plasmon resonance due to interaction between biological recognition element and target analyte. Owing to their high sensitivity and specificity, optical nanobiosensors are used in medical diagnostics^134^ and environmental monitoring^135^ and food safety.^136^ While optical biosensors rely on light-based transduction mechanisms, electrochemical biosensors utilize electrical signals to detect and quantify biomolecular interactions with high sensitivity. ***The electrochemical nanobiosensors*** detect changes in electrical properties^137,138^ like current, voltage, or impedance resulting from the interaction of the biological recognition element with the analyte. They may be classified based on their work principle as, a) amperometric sensors, b) potentiometric sensors, and impedimetric sensors. They are used in the measurement of glucose for diabetes management, water and food pathogen detection, and neurological research on neurotransmitters. Electrochemical biosensors often suffer from signal instability and interference in complex environments, which mechanical biosensors can overcome through direct force- or mass-based detection, offering higher robustness and stability. ***Mechanical nanobiosensors*** measure changes in mechanical properties after the target analyte binds to the sensor, such as mass, resonance frequency, or surface stress Cantilever-based sensors and QCM represent typical examples of such devices. These sensors can be used in medical diagnostics by detecting biomolecules involved in different diseases,^139^ for air quality monitoring, and in control of food safety and quality due to the precision measurement of minute mass changes. Mechanical biosensors can be limited by sensitivity and miniaturization challenges, which magnetic biosensors can overcome through noncontact, highly sensitive detection at the nanoscale ***Magnetic nanobiosensors*** make use of magnetic nanoparticles whose some magnetic properties change while they bind to the target analyte. Magnetic particle relaxation sensors and magnetic resonance sensors are falling under this category. They have been proved quite effective in detecting pathogens and toxins for biomedical applications, monitoring environmental contaminants, and point-of-care diagnostic devices due to their high sensitivity and possibility of working in complex biological environments as reported.^134^ Both mechanical and magnetic biosensors can face limitations in achieving real-time, label-free detection with ultrahigh sensitivity at the molecular level — a deficiency that plasmonic biosensors can overcome through their ability to amplify optical signals via localized surface plasmon resonance (LSPR), enabling rapid and highly sensitive analysis without the need for labels. ***Plasmonic nanobiosensors*** utilize the plasmonic properties of metallic nanoparticles, normally gold or silver, for detecting changes in their optical characteristics at the event of binding an analyte to the sensor surface. Popular examples are LSPR sensors and SERS sensors. These have wide applications in real-time monitoring of biomolecular interactions, chemical and biological species detection, and in environmental sensing due to the enhanced optical signal amplification.^135^ In comparison to previously discussed biosensors, Nanowires and nanobiosensors offer exceptional sensitivity and rapid response due to their high surface-to-volume ratio and nanoscale dimensions. **These biosensors** make use of nanowires or nanotubes as sensing elements; their electrical or optical characteristics are changed by binding with the target analyte. Examples include silicon nanowire sensors and carbon nanotube sensors. These sensors have been known to detect DNA, proteins, other biomolecules, and sense chemicals for environmental monitoring. In addition to high sensitivity these devices can be manufactured in miniature size enhancing its widespread application.^136^ Howver, Nanowire biosensors often face issues with nonspecific interactions and signal instability and Enzyme-based biosensors overcome this by offering high selectivity through specific biochemical reaction. ***Enzyme-based nanobiosensors*** make use of enzymes as biological recognition elements. An enzymatic reaction of the target analyte with the sensor gives rise to a detectable signal, typically through changes in either pH, conductivity, or optical properties.^137^ Glucose biosensors that make use of glucose oxidase and urea biosensors that make use of urease are examples. They find useful applications in medical diagnostics, environmental monitoring, and industrial process control especially where high selectivity to target analytes along with rapid results are required. Different types of nanobiosensors have special capabilities, making them quite appropriate for a wide range of applications in the medical, environmental, industrial, and safety domains. Their development continues to advance, promising even greater integration and functionality in the future.^138^

### 2.6 Key Materials and Technologies in Nanobiosensors

Nanobiosensors make use of a wide array of diversified, advanced materials and technologies for the improvement of sensitivity, specificity, and overall performance (Table 7). The most recent versions in the forefront include a) nanomaterials—nanoparticles, b) nanowires, c) nanotubes, and d) quantum dots—which show unique properties at a tiny scale. Among the materials considered for nanobiosensors, Gold nanoparticles are very much in use due to their very good biocompatibility and ability to provide a strong optical signal in plasmonic sensors. Likewise, nanomaterials of carbon-based classes, such as carbon nanotubes and graphene, are highly valued for their very high electric conductivity and large surface area making them suitable for electrochemical sensors^139^ (Figure 7 and Table 7). Further, quantum dots when integrated with advanced surface functionalization techniques, they become vital components in the development of high-performance nanobiosensors. These ***surface functionalization techniques*** allow specific attachment of biological recognition elements like antibodies, enzymes, or nucleic acids onto the surface aspect of nanomaterials.^140^ Functionalization of this nature can be realized through chemical modification or bioconjugation approaches, ensuring selective and stable binding of biological receptors to nanomaterials for the binding of target analytes. Surface functionalization techniques can lack structural precision and scalability.^141^ This deficiency can be overcome by microfabrication and nanofabrication technologies, which offer precise structural control and scalability for consistent and high-performance sensor development. Techniques like photolithography, electron-beam lithography, and soft lithography can be used to create nanobiosensors with high resolution and for precise integration of the nanomaterial into relevant responsive devices. In addition, ***Microfluidics*** is another technology which can be effectively integrated with nanobiosensors to form lab-on-a-chip systems.^142^ These chips combine several functions that are usually performed in a laboratory on one chip and hence result in a high output even with very small volumes. Microfluidic platforms enhance the efficiency and portability of nanobiosensors, making them suitable for point-of-care diagnostics and real-time environmental monitoring. In addition to the discussed technologies, ***Signal amplification and processing technologies*** can be used to enhance the detection capabilities of nanobiosensors.^143,144^ Techniques such as polymerase chain reaction (PCR) for nucleic acid amplification and signal amplification by reversible exchange (SABRE) in magnetic resonance sensors improve the sensitivity of these devices, allowing for the detection of low-abundance targets. Consequently, the integration of all such advanced materials and technologies has accelerated the development in nanobiosensors that enables applications in very diverse fields, from medical diagnostics^144,145^ and environmental monitoring^146^ to food safety^5^ and industrial process control.^147^ Continuous innovation in the area of nanomaterials and fabrication techniques will definitely result in improved capabilities and expanded applications for nanobiosensors in the future.

**Figure 7:**
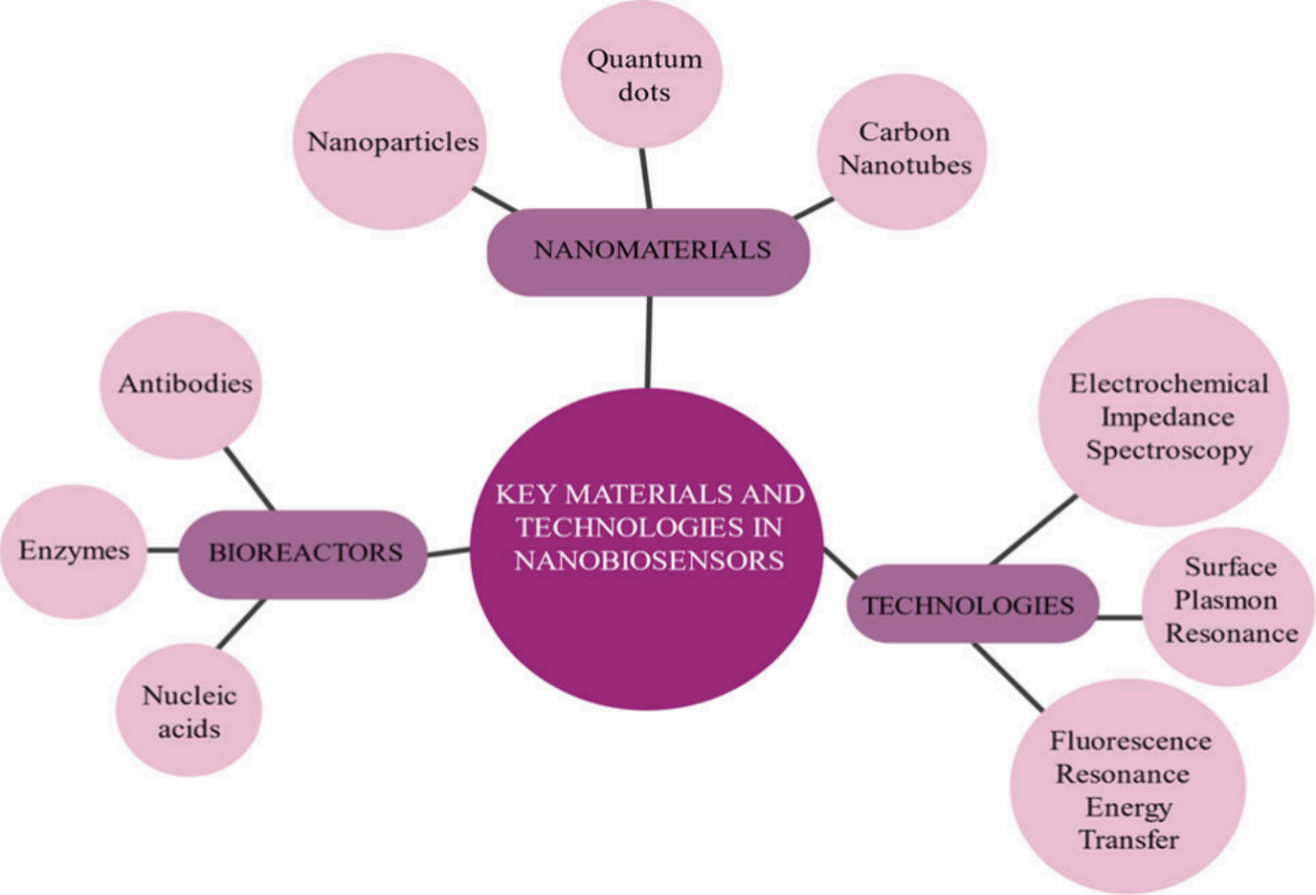
Key materials and technologies in nanobiosensors.

**Table 7:** Key Materials and Technologies in Nanobiosensors

**Material/Technology**	**Key Properties and Representative Applications**	**Scientific Description/Functional Mechanism**
Carbon Nanotubes (CNTs)	High electrical conductivity, large surface area; used in DNA, protein detection, and gas sensing.	One-dimensional carbon allotropes with excellent electrical and mechanical properties; enhance sensitivity and signal transduction in electrochemical and FET-based biosensors.
Gold Nanoparticles (AuNPs)	Distinctive optical properties due to SPR; applied in colorimetric sensors, SERS platforms, and electrochemical biosensors.	Metallic nanoparticles exhibiting localized surface plasmon resonance, enabling signal enhancement in optical and electrochemical detection methods.
Quantum Dots (QDs)	Size-dependent photoluminescence; used in multiplexed fluorescent biosensors and bioimaging.	Semiconductor nanocrystals with quantum confinement effects; allow tunable and stable fluorescence emission for labeling and detection.
Graphene and Graphene Oxide (GO)	High conductivity, mechanical strength, and functionalizability; used in sensors for glucose, DNA, and protein.	Two-dimensional carbon-based materials with exceptional charge mobility and tunable surface chemistry for biorecognition and signal amplification.
Magnetic Nanoparticles (MNPs)	Superparamagnetic properties; used in magnetic separation, biosensing, and immunoassays.	Typically composed of iron oxides; manipulated using magnetic fields for efficient target isolation and signal concentration in biosensing applications.
Electrochemical Sensing Platforms	High sensitivity, portability, and real-time capability; used in glucose monitors and biomarker detection.	Detect electroactive species by measuring current, potential, or impedance changes during biochemical reactions on electrode surfaces.
Optical Sensing Techniques	Sensitive detection via light absorption, fluorescence, or refractive index changes; used in pathogen and biomolecule sensing.	Utilizes optical signals for analyte detection, often integrated with nanomaterials to enhance signal output and specificity.
Surface Plasmon Resonance (SPR)	Label-free, real-time monitoring of biomolecular interactions; widely used in drug discovery and affinity studies.	Monitors changes in refractive index near a metal-dielectric interface to detect molecular binding events in real time.
Surface-Enhanced Raman Scattering (SERS)	Enables ultrasensitive molecular detection; used in diagnostics and chemical sensing.	Enhances Raman scattering of molecules adsorbed on rough metal surfaces (e.g., Au, Ag), providing unique vibrational fingerprints.
Label-Free Biosensing Approaches	Real-time, multiplexed detection without chemical labels; improves assay simplicity and speed.	Techniques like SPR and impedance spectroscopy detect analyte binding events directly by measuring intrinsic changes in physical properties.

## 3 Applications of Nanobiosensors in Structural Health Monitoring

The detailed applications are illustrated in Figure 8 and Table 8 and in sections below.

**Figure 8:**
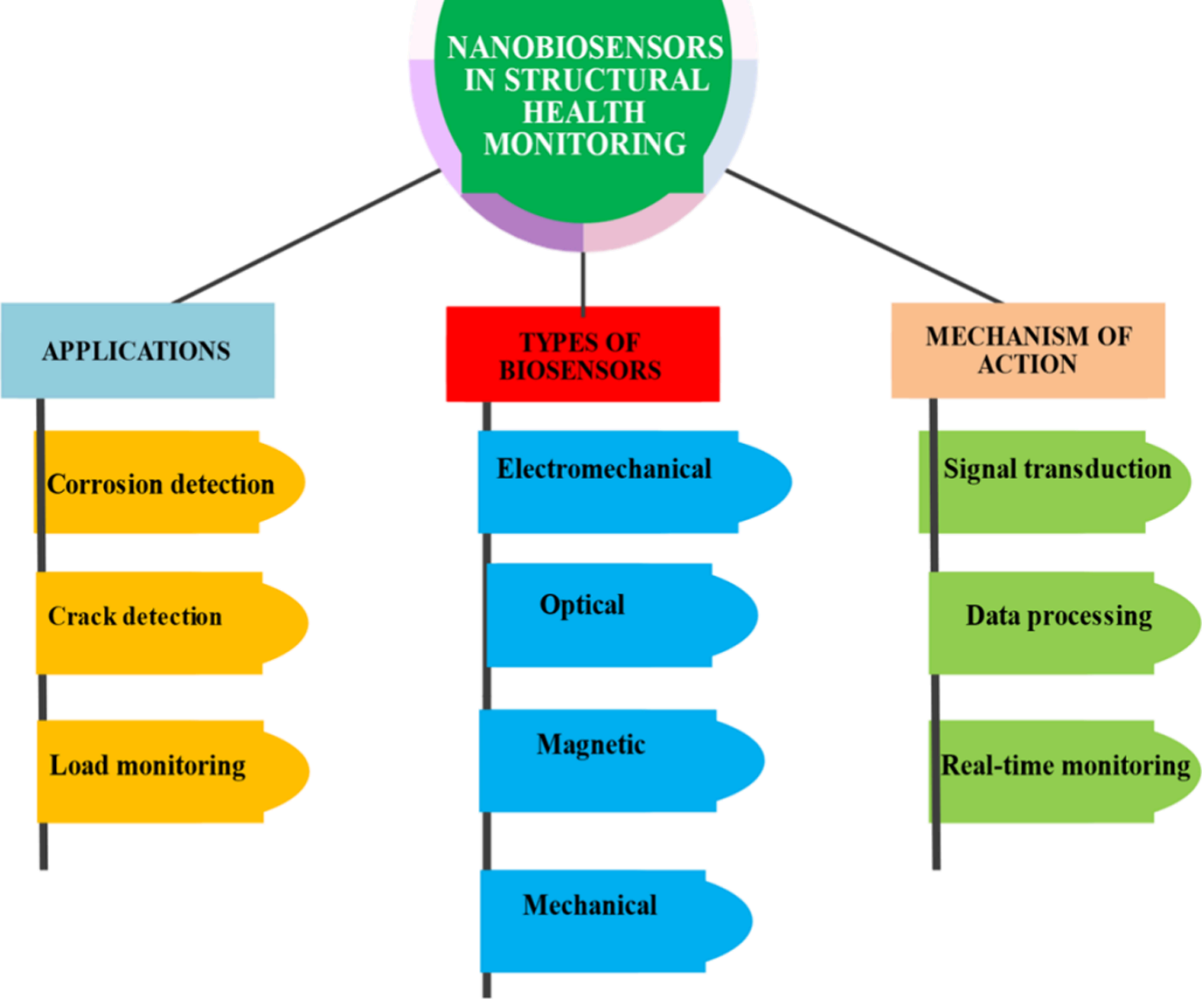
Nanobiosensors in structural health monitoring.

**Table 8:** Applications of Nanobiosensors in SHM

**Application**	**Description**
Crack Detection	Detection of microcracks and fractures in materials using nanobiosensors to identify early signs of structural failure.
Corrosion Monitoring	Monitoring corrosion levels in metallic structures, especially in harsh environments, using nanobiosensors sensitive to corrosion byproducts.
Stress and Strain Sensing	Measuring changes in stress and strain within structures using nanobiosensors to prevent failure due to excessive loads.
Temperature Monitoring	Monitoring temperature variations within structures to detect thermal stresses using nanobiosensors.
Fatigue Damage Assessment	Assessing fatigue damage in structures by monitoring accumulated damage over time with nanobiosensors.
Real-Time Monitoring	Providing real-time data on the structural integrity of buildings, bridges, and other critical infrastructure using embedded nanobiosensors.
Vibration Analysis	Analyzing vibrations in structures to detect anomalies and potential issues with the help of nanobiosensors.
Environmental Impact Assessment	Assessing the impact of environmental factors (e.g., humidity, pollution) on structural health using nanobiosensors.

Nanobiosensors can be integrated into structural materials, which monitor continuously for initiation of early damage. Molecular changes due to breakdown of compounds or formation of new compounds might indicate fatigue or degradation of material which can be easily tracked by nanobiosensors.^6^ Early identification of these changes makes it possible to plan maintenance, avoiding severe damage and potential failures and thereby promoting economy and sustainability. Corrosion is a destructive process which affects metals, reinforced concrete, and other components of the building. The nanobiosensors can be used for corrosion indication^148^ to detect the specific ions or molecules related to the initiation and progression of corrosion, for instance, chloride ions in concrete or sulfur compounds in metals. The continuous monitoring with these indicators would allow interventions, such as applying corrosion inhibitors and other protective measures, to lengthen the lifespan of the structure in the recommended way. Likewise, the health of a structure is heavily influenced by its surrounding environment. Nanobiosensors can measure environmental parameters like temperature,^149^ humidity,^149^ and pollutant levels.^150^ For instance, sensors detecting high levels of sulfur dioxide or carbon dioxide can indicate the risk of acid rain, which can accelerate the degradation of the materials. By providing real-time environmental data, these sensors help in assessing the impact of external conditions on structural health and guide appropriate maintenance strategies. Moreover, Nanobiosensors can be part of an integrated SHM system^6^ that utilizes the Internet of Things (IoT) and advanced data analytics. Such smart systems collect data from numerous sensors placed throughout a structure, analyze this data in real-time, and provide actionable insights.For example, in a bridge, an integrated system can monitor stress, strain, temperature, and corrosion indicators simultaneously, offering a comprehensive assessment of its health and predicting potential issues. Also, crack detection is an integral component in assessment of structural safety; Nanobiosensors can detect the initiation and growth of microcracks within a solid material. In this situation, the sensors are designed in such a way that they measure changes in local stress or strain within the material at the nanoscale to give a clear indication of when the cracks initiate and propagate. This is very important for critical structures like high-rise buildings, bridges, and dams, in which early detection of cracks can avert a possible catastrophic failure.^151^ Also, the assessment of structural behavior under different loads is necessary to ensure safety and performance of the structure.^151^ Nanobiosensors can transmit information on the changes in load distribution and intensity in real-time, giving information about the way a structure reacts to different stresses. For example, on bridges, they detect uneven load distributions connected with heavy traffic or another external force, enabling engineers to assess eventual damage to the integrity of the structures and consequently take measures.^152,153^ Moreover, in industrial settings, structures are often exposed to hazardous chemicals and gases. Nanobiosensors can detect the presence and concentration of such substances, ensuring safety and preventing structural damage. For instance, in chemical plants, sensors can monitor for leaks of corrosive gases like chlorine or hydrogen sulfide, providing early warnings and allowing for immediate corrective actions.^154^

### 3.1 Application of Nanobiosensors As Self-Healing Materials

Self-healing materials are designed to repair the damage on their own without human intervention, prolonging their lives and maintaining the structural integrity of the same. The most important advances in material science involve the embodiment of nanobiosensors into self-healing materials to track the health status of the material and tailor or enhance the self-healing process (Figure 9). Nanobiosensors embedded in the material continuously monitor its condition.^40^ They realize an early detection of microdamage in the form of cracks or voids by sensing changes in stress/strain or by detecting specific biomarkers indicative of damage. In this way, such precise detection enables the self-healing process to be triggered exactly where it is required, improving efficiency and effectiveness.^155^ This precise detection directly triggers the release of healing agents from embedded capsules as soon as microcracks emerge and Nanobiosensors are designed to release healing agents upon detection of damage

**Figure 9:**
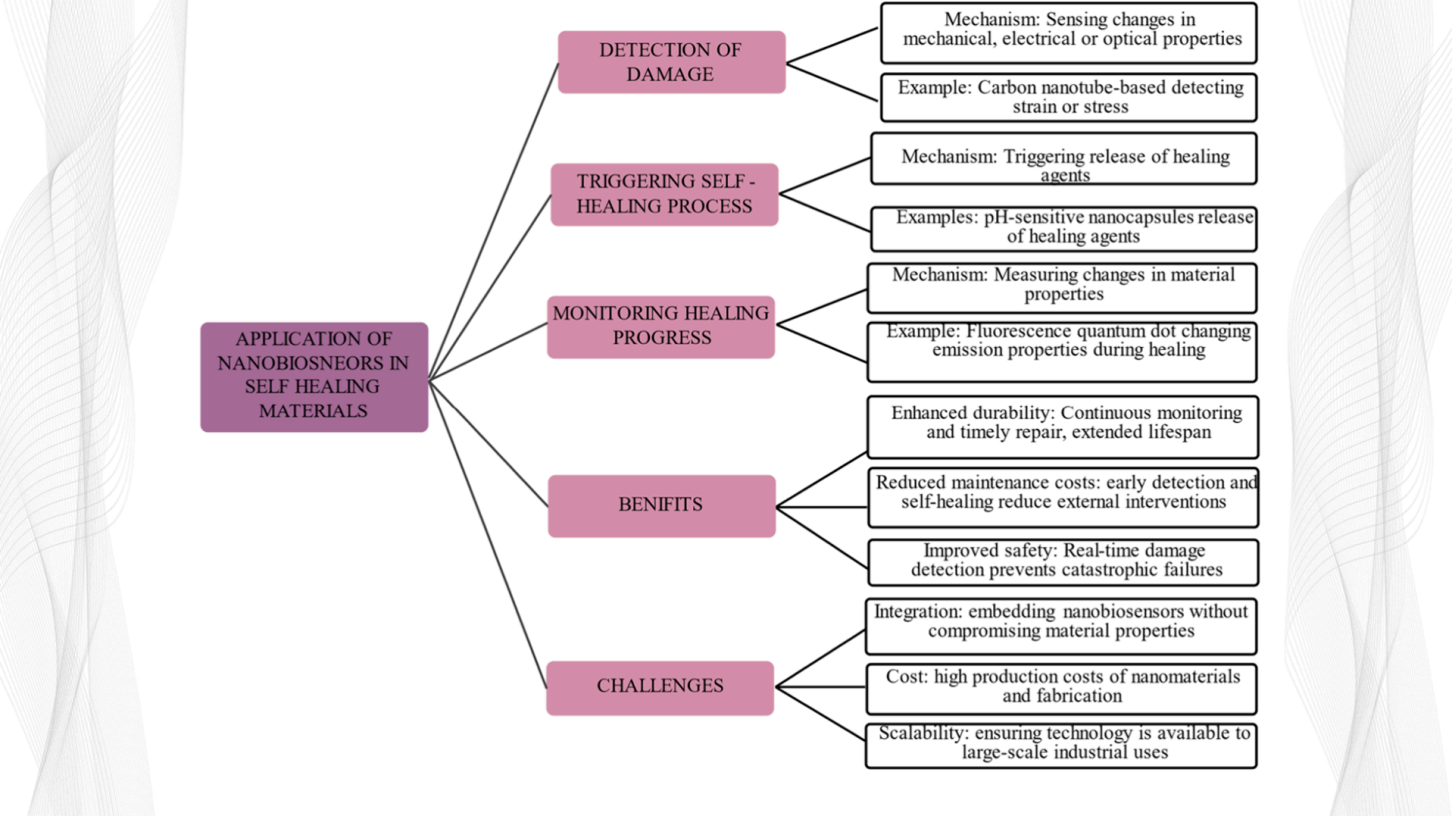
Application of nanobiosensors in self-healing

Usually, healing agents are encapsulated in the form of micro- or nanocapsules and distributed within the material. If a microcrack shows up, the nanobiosensors will sense it and send a signal to the capsules to deliver their content, which will then flow into the microcrack and harden, consequently healing the breakage.^156^ This smart response not only initiates healing but also allows nanobiosensors to precisely regulate the amount and rate of agent release for efficient repair. Also, Nanobiosensors can also control the extent of healing and the rate of release of healing/the healing agents. This will ensure that just the right quantity of material is used to heal damage, thereby averting costs by reducing waste and ensuring a complete repair. The progress of healing can also be monitored by such sensors, providing real-time feedback regarding its effectiveness.^157^ In advanced applications, nanobiosensors can constitute the basis of a fully autonomous healing response. Nanobiosensors can detect changes or stresses in the environment that will lead to subsequent damage and pre-emptively release protective agents. For example, upon exposure to UV radiation, nanobiosensors could release UV-blocking agents to prevent degradation in a polymer composite material. Furthermore, Nanobiosensors could be tailored to monitor different types of damage and dependent on damage could initiate one healing process or another. For example, according to a nano sensor, the changes may point toward chemical changes due to corrosion or to mechanical stresses due to cracking in a concrete structure. Dependent on the type of damage, these could release corrosion inhibitors or epoxy resin as healing agents. Tailored healing responses are paired with real-time monitoring, allowing nanobiosensors to repair damage while continuously assessing the structural health. These nanobiosensors integrated in this material will enable constant real-time monitoring of a structure’s health, providing important data on its condition and performance. All that can be used to schedule maintenance more effectively and predict the lifespan, reducing the potential for unplanned failures.

Nanobiosensors play a transformative role in structural health monitoring (SHM) by providing precise, real-time insights into the integrity and performance of structures. In corrosion detection, electromechanical and electrochemical nanobiosensors detect minute changes in the chemical environment, such as alterations in pH or ion concentration, indicating the onset of corrosion in reinforced concrete or steel components. For example, these sensors measure the electrochemical potential of steel reinforcement or detect chloride ion concentrations, enabling early intervention to prevent structural failure. Magnetic nanobiosensors are also employed for localized corrosion monitoring, where their high sensitivity to changes in the magnetic properties of materials helps identify corrosive processes at a nanoscale.^158^

In crack detection, optical nanobiosensors detect microlevel deformations or fractures by monitoring changes in refractive index or optical paths when cracks propagate in materials. Mechanical biosensors, based on piezoelectric principles, measure stress variations and acoustic emissions caused by crack growth, enabling real-time identification of damage zones.^159^ Similarly, load monitoring relies on the integration of nanobiosensors to measure the strain and stress distribution within structural elements under varying loads. Electromechanical sensors embedded in materials provide continuous monitoring by measuring changes in electrical conductivity or resistance correlated with load-induced deformation. These sensors, by combining advanced signal transduction, data processing, and real-time monitoring capabilities, ensure accurate and timely assessments of structural integrity, offering actionable insights that enhance safety, reduce maintenance costs, and extend the service life of critical infrastructure.^160^

Nanobiosensors offer transformative advantages when integrated into self-healing materials, significantly enhancing the performance, resilience, and sustainability of various structural systems. One of the primary benefits is the increased lifespan of materials, as continuous monitoring and autonomous repair of microdamages—enabled by nanobiosensor-based feedback mechanisms—allow for early intervention before minor issues evolve into critical failures. This proactive approach not only preserves structural integrity but also contributes to reduced maintenance costs, as it minimizes the need for routine inspections and labor-intensive repairs. By enabling real-time detection and localized healing, nanobiosensors also improve the overall safety and reliability of infrastructure, which is particularly crucial in high-stakes domains, such as aerospace, civil engineering, and transportation systems where undetected flaws can lead to catastrophic consequences. Moreover, the ability of these materials to self-repair and thereby extend their operational life contributes to greater sustainability as it reduces material consumption, lowers the frequency of replacements, and supports circular economy principles in construction and manufacturing sectors. Thus, the convergence of nanobiosensors and self-healing materials represents a forward-looking strategy for building more durable, cost-efficient, and environmentally responsible structures.

However, certain challenges are faced by nanobiosensors as self-healing materials like material compatibility. It is crucial to ensure that nanobiosensors do not affect the host material. The devices should not interfere with the material’s original properties and its capability of self-healing. Researchers are also working on sensor designing and development compatible for most self-healing materials. Further, functionality of the nanobiosensors must be maintained over the entire lifetime of the material, which in extreme conditions poses a difficulty. To have improved durability and reliability in such sensing devices, future progress in relation to nanotechnology and materials science is needed. Moreover, the cost and scalability aspect are one of the pressing issues in nnaobiosensors. Producing nanobiosensor-enabled self-healing materials at scale and at a reasonable cost is a significant challenge. Developing cost-effective manufacturing processes and materials will be the key to widespread adoption. In addition, real-time data analytics and storage solutions for data generated by nanobiosensors are complex. From a practical point of view, making these systems compatible and user-friendly and integrating them into existing infrastructure are quite important. Nanobiosensors will play a very important role in the development of self-healing materials through early damage detection, triggering healing mechanisms, and real-time monitoring, which enhance the rate of healing. Challenges still exist, but current research and technological advances keep opening new avenues for the wider application of such innovative materials to reach safer, more durable, and sustainable structures in many industries.

### 3.2 Nanobiosensors in Real-Time Structural Integrity Assessment

Nanobiosensors, due to their high sensitivity and specificity, find increasing application in the integration of systems for real-time assessment of structural integrity. Such an application is relevant to enforce the safety, reliability, and durability of many structures like buildings, bridges, dams, and pipelines. The following is a comprehensive explanation with details of how nanobiosensors are applied in this regard (Figure 7). Nanobiosensors offer a comprehensive and dynamic approach to structural health monitoring by enabling real-time, continuous assessment of material performance and environmental impact. When embedded directly into structural components, these sensors provide **continuous monitoring** by detecting subtle variations in parameters such as stress, strain, temperature, humidity, and presence of chemical or biological agents. This embedded capability is especially critical for infrastructure like bridges, where nanobiosensors can detect early signs of **metal fatigue or corrosion**, allowing for predictive maintenance and timely intervention before failure occurs.^161^

A particularly valuable function of these sensors lies in their ability to detect **microcracks and stress** at the nanoscale—an early indicator of larger structural degradation. In concrete structures, for example, fluctuations in temperature or sustained mechanical loading can induce microcracking, which may not be visible through conventional inspection methods. Nanobiosensors respond to these minute changes in the material matrix, enabling pre-emptive action to mitigate further deterioration.^162^ In parallel, **corrosion monitoring** using nanobiosensors enhances long-term durability by identifying corrosive agents such as chloride ions within reinforced concrete or metallic infrastructure. These sensors monitor localized electrochemical changes associated with corrosion onset and progression. When implemented in fluid distribution systems, nanobiosensors can act as internal sentinels, alerting operators the moment corrosive conditions develop, and triggering automated alarms upon crossing critical thresholds.^163^ Beyond internal structure surveillance, nanobiosensors are also effective in **environmental sensing**, tracking external variables that influence structural longevity. Parameters such as the ambient temperature, humidity, pH, and pollutant levels can accelerate degradation if left unmanaged. In coastal zones, for instance, nanobiosensors can detect elevated salt concentrations in the air—data that informs corrosion control strategies and adaptive maintenance schedules for steel infrastructure.^164^ IOT is being actively connected with nanobiosensors^165^ and their compatibility with digital infrastructure further allows **integration with smart systems**, particularly Internet of Things (IoT)-enabled structural health monitoring (SHM) platforms. These integrated systems collect and process data from numerous nanobiosensors in real time, providing a holistic view of a structure’s condition. In high-rise buildings, for example, nanobiosensors can be utilized to assess load distribution patterns, identify early signs of fatigue, and trigger predictive maintenance through AI-driven analytics.

Nanobiosensors also contribute to **biological agent detection**,^166^ offering vital early warnings against microbial threats that can compromise structural integrity or health safety. The growth of mold, algae, or bacteria on structural surfaces or within HVAC systems can be quickly detected by biosensors tailored to identify specific biological markers. This facilitates prompt remediation and preserves the indoor environmental quality.

Moreover, their role in **chemical and gas detection** ^167^ is indispensable in high-risk settings such as industrial plants or storage facilities. Nanobiosensors can precisely detect hazardous gases such as ammonia or hydrogen sulfide, which, if undetected, may not only corrode infrastructure but also pose serious safety risks. The sensors’ rapid response enables immediate containment actions, thereby safeguarding both the structural framework and human operators.

Altogether, the integration of nanobiosensors into modern infrastructure enables a shift from reactive to proactive structural health management. By providing precise, localized, and real-time data on both internal and external threats, these sensors enhance the reliability, safety, and sustainability of complex built environments (Figure 10).

**Figure 10:**
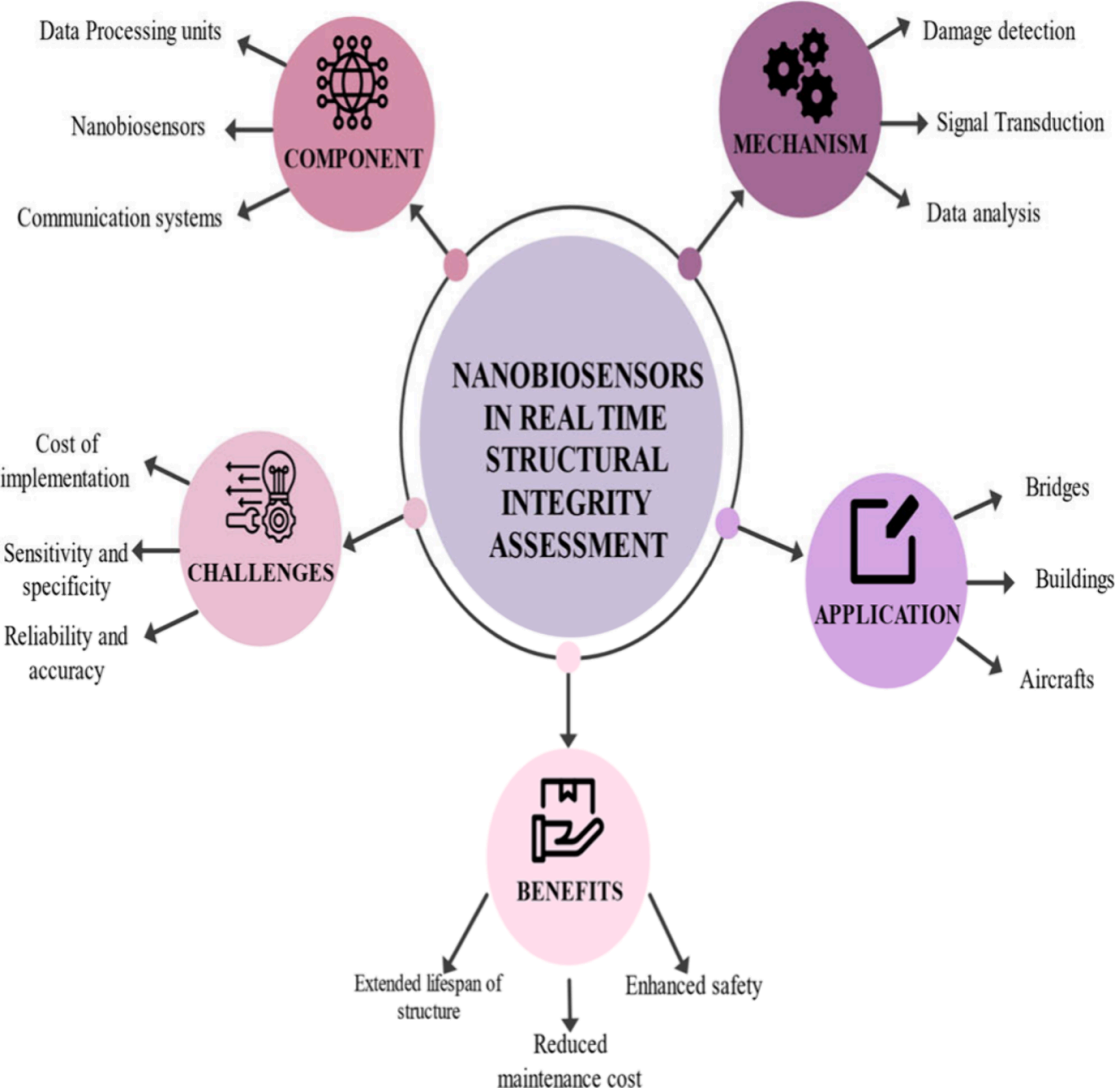
Nanobiosensors in structural integrity assessment.

### 3.3 Advantages and Challenges of Nanobiosensors in Real-Time Structural Integrity Assessment

Nanobiosensors offer a promising approach to structural health monitoring^168^ by combining high sensitivity and specificity with the ability to detect minute molecular changes that may indicate early signs of damage. Their capability for real-time monitoring ensures continuous data collection, allowing for immediate responses to structural anomalies. This early detection plays a critical role in enabling preventive maintenance and reducing the risk of sudden structural failures. Furthermore, nanobiosensors support comprehensive assessment by providing a detailed, holistic view of the material or structure’s condition, encompassing physical, chemical, and sometimes even biological parameters. One of the standout features of nanobiosensors is their ability to trigger targeted healing responses such as releasing corrosion inhibitors or epoxy resins based on the type of damage detected. Simultaneously, they enable real-time condition tracking, ensuring that structural integrity is not only restored but also constantly assessed. These smart systems can even regulate the rate and quantity of healing agent release, optimizing resource use and ensuring efficient self-repair mechanisms.

Despite these challenges, nanobiosensors represent a transformative advancement in structural health monitoring. Their integration into the critical infrastructure could drastically enhance safety, reliability, and maintenance efficiency. As nanotechnology continues to evolve, the convergence of miniaturized sensors, smart materials, and self-healing mechanisms positions nanobiosensors as a cornerstone in the future of intelligent infrastructure systems.

### 3.4 Environmental Monitoring and pollution detection

Nanobiosensors have properties typical of both nanomaterials and biological recognition elements and, therefore, contribute significantly toward environmentally remedial applications.^169^ Indeed, the developed sensors can be extremely sensitive and selective, allowing trace detection of pollutants—making them highly effective tools for both environmental monitoring and cleanup efforts. Their ability to detect and respond to specific contaminants in real time has revolutionized several domains of environmental remediation, as summarized in Figure 11. However, despite their transformative potential, several critical challenges continue to hinder the widespread application of nanobiosensors in structural systems. One of the foremost difficulties lies in **sensor integration and scalability**, particularly when attempting to embed these sensors across large-scale or geometrically complex infrastructures. Achieving seamless integration demands sophisticated strategies that ensure uniform sensor distribution, robust power supply management, and reliable data transmission across expansive networks. This necessitates the development of adaptive interfacing protocols and advanced wireless communication systems capable of maintaining real-time connectivity without signal loss or degradation.Equally important is the challenge of **durability and longevity**, as nanobiosensors deployed in real-world infrastructure must operate reliably over extended periods while enduring harsh environmental conditions. Temperature fluctuations, high humidity, exposure to corrosive chemicals, and mechanical stress can all compromise sensor performance. Ongoing research is focused on developing protective coatings, resilient packaging materials, and self-healing components to enhance the environmental stability and operational lifespan of these sensors, ensuring their sustained accuracy in the field. Another significant concern is **data management and analysis**, as nanobiosensors generate vast streams of high-resolution, high-frequency data that require real-time processing. Transforming this raw data into meaningful insights demands powerful analytics platforms equipped with advanced algorithms for pattern recognition, anomaly detection, and predictive modeling. Additionally, scalable cloud storage, intuitive user interfaces, and interactive visualization tools are essential to facilitate informed decision-making by engineers and infrastructure managers.

**Figure 11:**
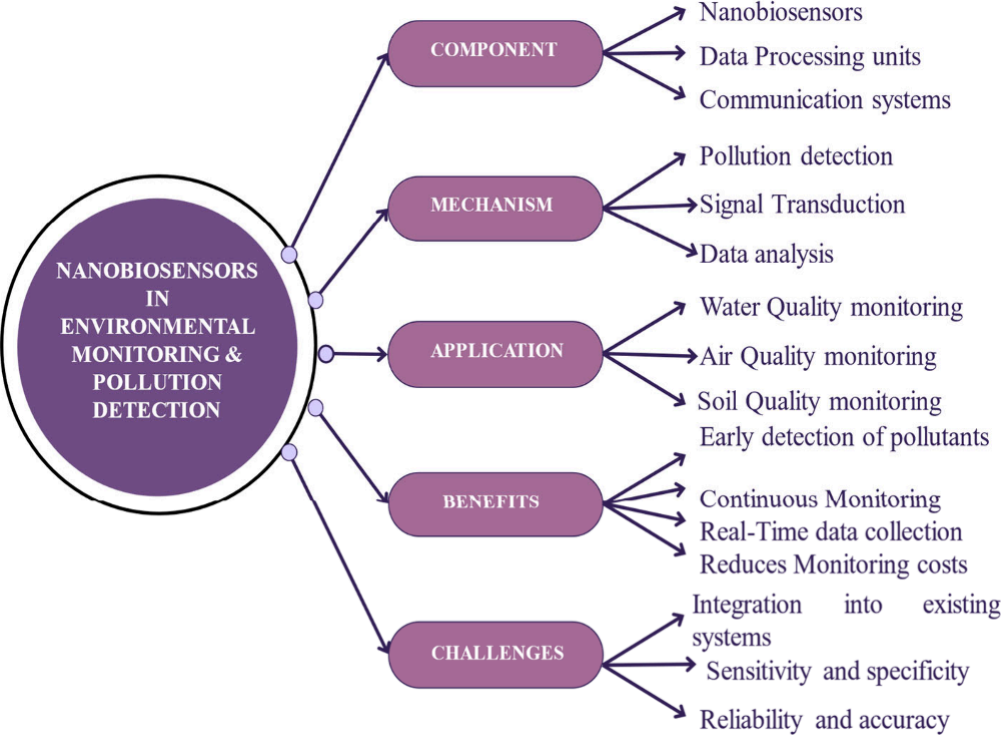
Nanobiosensors in environment pollution monitoring.

Lastly, **cost considerations** continue to pose a major barrier to mass deployment. The fabrication and deployment of nanobiosensors currently involve high production costs due to the complexity of materials, precision manufacturing, and integration procedures. However, recent advances in low-cost nanomaterial synthesis, scalable fabrication methods, such as roll-to-roll printing, and modular sensor design are steadily reducing these costs. As these technological innovations mature, nanobiosensors gradually become more economically viable, bringing their widespread adoption within reach.

One of the key applications is in the detection of heavy metals such as lead, mercury, and cadmium in water bodies. Upon functionalization with specific biological receptors, nanoparticles within the biosensors bind to these metals, altering their optical or electrical properties to signal contamination. In a similar vein, organic compounds such as pesticides, herbicides, and industrial solvents can be detected through recognition of their molecular structures or metabolic byproducts, ensuring early identification of chemical hazards.

Nanobiosensors are also pivotal in air quality monitoring.^170–172^ They can detect volatile organic compounds (VOCs), carbon monoxide, nitrogen oxides, and sulfur dioxide, enabling real-time assessments that support prompt risk mitigation. For example, in indoor environments, these sensors can monitor VOCs from sources such as paints and cleaning agents, ensuring safe air for inhabitants. In urban settings, they help track pollutants from vehicles and industries, contributing to regulatory enforcement and public health protection.

In the context of water quality monitoring, nanobiosensors are deployed to detect microbial contaminants,^173–175^ chemical pollutants,^176–179^ and changes in water chemistry in both freshwater and marine systems. Their precision allows for applications such as pathogen detection—identifying bacteria, viruses, and protozoa in drinking and recreational waters—and nutrient monitoring, measuring levels of nitrates and phosphates that could lead to eutrophication and algal blooms. Also, Contaminated soil remains a significant environmental concern.^169^ Nanobiosensors can evaluate contamination levels, monitor cleanup progress, and verify the success of remediation activities. For instance, they can detect hydrocarbons from oil spills or pesticide residues in agricultural soils, helping to tailor bioremediation efforts and prevent further ecological damage.

These sensors can also be seamlessly integrated into bioremediation systems, providing real-time data on the metabolic activity of pollutant-degrading microbes. This ensures optimal environmental conditions and enhances the efficiency of the process. Examples include oil spill cleanup, where sensors monitor hydrocarbon degradation by bacteria, and heavy metal remediation, guiding the deployment of metal-absorbing microbes or plants.

In wastewater treatment plants, nanobiosensors monitor both contaminant presence and process efficiency. This ensures that treated water released into the environment meets the safety standards. For example, nutrient removal processes benefit from sensors that track ammonia, nitrate, and phosphate levels, while pathogen monitoring ensures disinfection efficacy before discharge.

#### 3.4.1 Advantages of Nanobiosensors in Environmental Remediation

Nanobiosensors bring several advantages to environmental remediation. They exhibit high sensitivity and specificity, enabling the detection of pollutants at trace concentrations with an impressive accuracy. Their real-time monitoring capability ensures continuous tracking and an immediate response to environmental threats. Their compact design makes them portable and easily deployable in the field, facilitating on-site assessments in remote or complex locations. Additionally, the cost-effectiveness of early detection and targeted remediation helps to minimize both the environmental impact and overall cleanup expenses.

#### 3.4.2 Challenges and Future Directions in Use of Nanobiosensors in Environmental Remediation

Despite their immense potential, the widespread adoption of nanobiosensors in environmental applications faces multiple challenges. One of the foremost issues is sensor durability, as nanobiosensors deployed in the environment must withstand fluctuating temperatures, humidity, mechanical stress, and exposure to corrosive chemicals. Extensive research is underway to enhance the structural robustness and long-term stability of these sensors to ensure reliable performance in harsh settings.

Another critical challenge lies in data management. Nanobiosensors generate large volumes of high-resolution, continuous data that require sophisticated analytical tools for real-time processing, storage, and interpretation. Developing user-friendly interfaces and intuitive visualization platforms is essential to help end-users, including environmental engineers and policy makers, translate these complex data into actionable insights.

Integrating these advanced sensors with existing environmental monitoring and remediation infrastructures also presents difficulties. Compatibility with legacy systems and seamless data exchange are crucial to ensure the practical implementation of nanobiosensor-based technologies. Without such integration, the full potential of these systems cannot be realized within the current frameworks.

Furthermore, regulatory scrutiny and public perception remain significant barriers. The use of nanotechnology in the environment often raises concerns about safety, ecological impact, and long-term consequences. For nanobiosensors to gain widespread acceptance, it is essential to demonstrate their safety, effectiveness, and environmental benefits clearly. Transparent validation, compliance with regulations, and proactive communication with stakeholders will play key roles in overcoming these concerns. In conclusion, nanobiosensors offer a powerful and versatile platform for environmental remediation. Their high sensitivity, specificity, and ability to provide real-time data make them ideal for applications ranging from air and water quality monitoring to soil remediation and bioremediation enhancement. While challenges related to durability, data handling, system integration, and public trust remain, rapid advancements in nanotechnology, materials science, and sensor design are steadily addressing these limitations. As these hurdles are overcome, nanobiosensors are expected to play a transformative role in enabling cleaner, safer, and more sustainable environments in the near future.^170^

### 3.5 Nanobiosensors in Smart and Sustainable Infrastructure

Nanobiosensors have increasingly taken center stage in the drive toward smart and sustainable infrastructure through a blend of nanotechnology with biological sensing elements. These advanced sensors provide highly accurate real-time monitoring across a wide range of parameters, significantly enhancing efficiency, safety, and sustainability in both urban and rural environments. One of the most impactful applications is in real-time structural health monitoring, where nanobiosensors embedded in construction materials can continuously track stress, strain, cracks, and other anomalies at the molecular level. This enables early detection and intervention, preventing catastrophic failures. For instance, in bridges and highways, these sensors detect microcracks and material fatigue, sending alerts for timely maintenance and ensuring long-term structural integrity.

In addition to structural monitoring, nanobiosensors contribute to energy efficiency and optimization in buildings and industrial facilities. By monitoring environmental conditions such as temperature, humidity, and air quality, they allow systems like HVAC to automatically adjust, reducing energy consumption without compromising comfort. In smart buildings, this translates to intelligent control of heating, cooling, and lighting based on real-time occupancy and environmental data. Moreover, the integration of nanobiosensors into infrastructure supports environmental monitoring and remediation. They enable constant assessment of environmental parameters to maintain ecological balance and ensure compliance with environmental regulations. For example, in waste management facilities, these sensors assist in in-line detection and sorting, monitoring landfill conditions, and optimizing recycling processes. In water systems, nanobiosensors offer real-time alerts on the presence of contaminants—including heavy metals, pathogens, and chemical pollutants—thus safeguarding drinking water quality and improving wastewater treatment efficiency.^171^

Nanobiosensors are also proving valuable in smart waste management. In landfills, they monitor parameters like methane levels and leachate composition, helping prevent environmental contamination and improving decomposition management. Similarly, in air quality control, these sensors enable real-time detection of particulate matter, volatile organic compounds, and greenhouse gases, particularly in urban areas. A network of such sensors can provide detailed air quality maps, empowering authorities to regulate traffic, industrial emissions, and other pollution sources to protect public health.

Their role extends further into agriculture, where nanobiosensors are transforming traditional farming into smart, data-driven agriculture. By offering real-time insights into soil nutrients, moisture levels, and pH, they help farmers apply fertilizers and irrigation more precisely, minimizing environmental impact and maximizing crop yields.^100^ In the realm of sustainable energy, nanobiosensors contribute to the efficient operation of renewable energy systems. For example, in solar panels, sensors detect dirt accumulation or structural damage, prompting timely cleaning and repair to maintain optimal performance. Similarly, they can be used in wind turbines and other renewable devices to monitor system health and to ensure operational efficiency.

Perhaps one of the most transformative aspects of nanobiosensors is their seamless integration with Internet of Things (IoT) platforms and advanced data analytics. This fusion enables holistic monitoring, predictive maintenance, and intelligent decision-making across various infrastructure elements. In smart cities, such integration allows for real-time tracking of traffic flow, energy consumption in buildings, environmental fluctuations, and public safety concerns. Collectively, this forms a comprehensive overview of urban dynamics, supporting proactive governance and sustainable development.^172^ As nanobiosensor technology continues to evolve, its potential to redefine infrastructure systems and drive global sustainability grows ever more significant.

#### 3.5.1 Advantages and Challenges of Nanobiosensors in Smart and Sustainable Infrastructure

Nanobiosensors offer several distinct advantages that make them highly suitable for smart and sustainable infrastructure. Their high sensitivity and specificity allow for the detection of minute changes and trace concentrations of specific substances, enabling the early identification of structural or environmental issues before they escalate. The capability for continuous real-time data collection provides immediate feedback, supporting timely interventions and proactive management. Additionally, nanobiosensors contribute to cost-effectiveness by optimizing resource usage and reducing maintenance and operational expenses through early fault detection and precision monitoring. From a sustainability standpoint, their use enhances overall resource efficiency, minimizes waste, and reduces environmental impact, making them valuable tools for eco-conscious development and infrastructure management.

However, the practical implementation of nanobiosensors in infrastructure systems comes with several challenges. Ensuring durability and longevity is paramount, as these sensors must maintain functionality under a wide range of environmental conditions over extended periods. Current research is focused on improving sensor robustness to withstand harsh factors such as humidity, temperature fluctuations, and physical stress.^173^ Another major concern is data management and security. The large volume of data generated by these sensors necessitates advanced data processing systems and secure communication protocols to protect sensitive and critical information.^174^ Furthermore, integration and scalability remain significant technical and logistical hurdles. Incorporating nanobiosensors into existing infrastructure and deploying them at scale requires standardized protocols, interoperability, and cost-effective production methods to ensure broad and seamless adoption.^175^ Equally important are public acceptance and development of suitable regulatory frameworks. Public awareness and trust in the safety and benefits of nanotechnology in infrastructure are essential for successful implementation. Therefore, clear communication, transparency in deployment, and adherence to well-defined regulations are crucial to gaining the trust of stakeholder confidence and regulatory approval.

In summary, nanobiosensors are poised to revolutionize smart and sustainable infrastructure by offering precise, real-time monitoring capabilities that enable proactive and intelligent management of structural health, environmental parameters, and energy efficiency. Although current challenges related to durability, data handling, system integration, and public perception exist, rapid advancements in nanotechnology, sensor engineering, and digital infrastructure are steadily addressing these concerns. As these innovations continue to mature, nanobiosensors will play a pivotal role in creating safer, more resilient, and environmentally sustainable urban and rural ecosystems.

### 3.6 Nanobiosensors in Biomedical Applications: Roles, Benefits, and Advancements

Nanobiosensors combine the principles of nanotechnology and biology to create highly sensitive, specific, and responsive platforms capable of detecting biological molecules and processes at unprecedented precision. These devices leverage the unique physicochemical properties of nanomaterials—such as high surface-to-volume ratio, tunable surface chemistry, quantum effects, and enhanced electron mobility—alongside biological recognition elements such as enzymes, antibodies, aptamers, or DNA probes to achieve selective and real-time detection. Their compactness, biocompatibility, and scalability make them indispensable tools in a wide range of biomedical applications, from disease diagnosis to personalized therapy.

One of the most transformative uses of nanobiosensors is in disease diagnosis and monitoring, where their ultrasensitivity enables the detection of biomarkers at femtomolar or even attomolar concentrations. This allows for the early stage identification of diseases long before clinical symptoms appear. For example, in cancer diagnostics, nanobiosensors can detect circulating tumor DNA (ctDNA), cancer-specific RNA transcripts, or overexpressed proteins in blood or biopsy samples, helping clinicians make accurate and timely decisions tailored to individual patients.^176^ Similarly, in diabetes care, wearable nanobiosensors integrated with microneedle patches can continuously monitor glucose levels in interstitial fluid, offering real-time glycemic control without the need for frequent finger-prick tests.^177^

These sensors are also revolutionizing point-of-care testing (POCT) by enabling rapid, accurate, and decentralized diagnostics. Unlike traditional diagnostics, which require lab infrastructure, nanobiosensors can provide on-site results within minutes. For instance, portable devices equipped with nanostructured surfaces functionalized with antibodies or aptamers can detect infectious agents such as HIV, influenza, or SARS-CoV-2 directly from saliva, blood, or nasal swabs—making them especially useful in rural or resource-constrained settings.^178^

Their integration into personalized medicine has also become a game-changer. By accurately detecting genetic or proteomic biomarkers, nanobiosensors facilitate treatment regimens that are customized to the molecular profile of individual patients. For example, real-time drug monitoring using electrochemical nanobiosensors can help maintain optimal plasma drug concentrations, especially for narrow therapeutic index medications such as antiepileptics or chemotherapy agents, thus improving efficacy and reducing toxicity.^179^

The potential of nanobiosensors extends to real-time health monitoring, particularly through wearable technologies. Flexible, skin-adherable sensors embedded with carbon nanotubes or graphene-based materials can continuously track cardiac activity, respiratory rate, hydration, or electrolyte balance, offering clinicians and users a comprehensive view of their physiological status. These wearables not only enhance patient compliance but also facilitate remote health surveillance, making healthcare more accessible and preventive.^180^

In the field of tissue engineering and regenerative medicine, nanobiosensors serve as intelligent tools for monitoring the microenvironment during cell culture or scaffold development. For instance, biosensors can track oxygen levels, pH, or metabolite concentrations to ensure optimal conditions for tissue growth. In more advanced applications, they can detect stem cell differentiation markers in real-time, allowing researchers to fine-tune culture conditions and improve the reliability of regenerative therapies.^181^

In drug discovery and development, nanobiosensors are employed in high-throughput screening platforms. They allow pharmaceutical researchers to rapidly assess the interactions between thousands of drug candidates and their biological targets. By utilizing techniques such as localized surface plasmon resonance (LSPR) or electrochemical impedance spectroscopy (EIS), these sensors can detect binding events without the need for labeling, significantly accelerating the drug screening pipeline and improving hit-to-lead conversion rates.^182^

Nanobiosensors also find crucial applications in detecting environmental toxins and pathogens that pose public health risks. In food safety, for instance, biosensors can be used to detect contaminants, such as *E. coli*, *Salmonella*, or pesticide residues, ensuring product safety from farm to table. In water and air monitoring, they can provide early warnings for biological hazards, chemical leaks, or bioterrorism threats.^183^

Moreover, the integration of nanobiosensors in implantable medical devices is reshaping chronic disease management. These implantable sensors can monitor physiological signals and respond to therapeutic actions autonomously. For instance, glucose-monitoring implants can be coupled with insulin pumps to create closed-loop systems for managing diabetes, automatically adjusting insulin delivery based on real-time glucose readings.^184^ Similarly, biosensors in cardiac pacemakers or neurostimulators can help personalize therapy based on the patient’s current condition.

#### 3.6.1 Advantages of Nanobiosensors in Biomedical Applications

Nanobiosensors offer several key advantages that make them invaluable in modern healthcare. Their high sensitivity and specificity allow for detection of extremely low concentrations of analytes, enabling early diagnosis and precision medicine. They provide rapid and real-time analysis, which is essential for critical care and emergency scenarios. Their miniaturized form allows integration into wearable and portable devices, enhancing accessibility and patient compliance. Furthermore, they are cost-effective, often reducing the need for bulky laboratory equipment and frequent clinical visits. Perhaps most importantly, nanobiosensors enable a high degree of personalization, providing molecular insights that allow clinicians to tailor therapies and interventions with unprecedented accuracy.As biomedical research and nanotechnology continue to converge, the role of nanobiosensors is expected to expand dramatically, heralding a new era of proactive, precision-driven, and patient-centered healthcare.

### 3.7 Challenges and Future Directions applications of Nanobiosensors in Biomedical Applications

#### 3.7.1 Biocompatibility and Safety

Ensuring the biocompatibility of nanobiosensors and their long-term safety in the human body is crucial (Tables 9–11). Research is focused on developing nontoxic, stable, and biocompatible nanomaterials. The biocompatibility concerns of nanobiosensors extend beyond initial material toxicity to include immune activation and chronic inflammation. Nanoparticles may trigger foreign body responses, leading to fibrous encapsulation and impaired sensor function. Additionally, degradation products, such as metal ions or polymer fragments, can accumulate and exert cytotoxic or immunogenic effects over time. Long-term in vivo studies are essential to assessing systemic tolerance and functional stability. Pre-existing anti-PEG antibodies have been detected in individuals with no prior exposure to PEGylated drugs, with early studies reporting a prevalence of 0.2% to 25% using hemagglutination assays.^185^ Subsequent studies confirmed this finding but showed wide variability due to assay format and sensitivity differences.^185,186^ Direct binding assays (e.g., ELISA) detect anti-PEG IgG more accurately than bridging assays, which often underestimate responses.^187^ Recent use of standardized humanized anti-PEG IgM/IgG antibodies has improved cross-study comparisons.^188^ In mice, B-1 cells naturally secrete anti-PEG antibodies, and similar human B-cell subsets may exist.^189,190^ Genetic predispositions linked to immunoglobulin heavy-chain variants may also influence anti-PEG IgM formation.^191^ Environmental exposure via PEG-containing products and inflammation at dermal sites^192,193^ may further induce these antibodies.^194^ A study on 2,404 healthy Han Chinese donors revealed a 43.1% overall prevalence, with 26.4% positive for IgM, 25% for IgG, and 8.3% for both.^188^ Anti-PEG IgG prevalence and concentration declined with age, while IgM remained age-independent. Concentrations of IgM and IgG ranged from 0.2–57 μg/mL and 0.3–238 μg/mL, respectively, with higher antibody levels observed in female donors. The high prevalence of anti-PEG antibodies indicates a strong immunogenicity from repeated exposure to PEGylated products. Elevated antibody levels in females may result from enhanced dermal absorption or hormonal influences on immunity. The decline of IgG with age suggests reduced memory B-cell activity, while stable IgM levels imply continuous naïve B-cell engagement. These patterns raise safety concerns, such as accelerated blood clearance (ABC) and hypersensitivity to PEGylated drugs. Screening patients for anti-PEG isotypes could optimize drug design and clinical outcomes in nanomedicine. Similar studies have been reported in other research works also.^195,196^

**Table 9:** Clinically Used Pegylated Protein Drugs^197^

**brand name**	**common name**	**component**	**source**	**type**	**PEG (kDa)**	**PEG number**	**disease**	**year approved**
Adagen	pegademase	adenosine deaminase	bovine	enzyme	5	11–17	severe combined immunodeficiency	1990
Oncaspar	pegaspargase	l-asparaginase	*E. coli*	enzyme	5	69–82	leukemia	1994
PEG-Intron	PEG interferon	interferon alfa-2b	human	cytokine	12	1	hepatitis C	2001
Neulasta	pegfilgrastim	G-CSF	human	cytokine	20	1	neutropenia	2002
Pegasys	peginterferon alfa-2a	interferon alfa-2b	human	cytokine	40	1 (branched)	hepatitis	2002
Somavert	pegvisomant	antagonist (GHR)	human	protein	5	4–6	acromegaly	2003
Mircera	PEG-epoetin beta	epoetin beta	human	protein	30	1	anemia	2007
Cimzia	certolizumabpegol	anti-TNFα Fab	human	antibody	40	1 (branched)	rheumatoid arthritis	2008
Krystexxa	pegloticase	uricase	porcine	enzyme	10	9	gout	2010
Sylatron	peginterferon alfa-2b	interferon alfa-2b	human	cytokine	12	1	melanoma	2011
Lonquex	lipegfilgrastim	G-CSF	human	cytokine	20	1	neutropenia	2013
Plegridy	peginterferon beta-1a	interferon beta-1a	human	cytokine	20	1	multiple sclerosis	2014
Adynovate	PEG-antihemophilic factor	factor VIII	human	protein	20	1 (branched)	hemophilia A	2015
Rebinyn	coagulation factor IX	factor IX	human	protein	40	1	hemophilia B	2017
Jivi	PEG-antihemophilic factor	factor VIII	human	protein	60	1 (branched)	hemophilia A	2018
Fulphila	pegfilgrastim	G-CSF	human	cytokine	20	1	neutropenia	2018
Revcovi	elapegademase	adenosine deaminase	bovine	enzyme	5	13	severe combined immunodeficiency	2018
Asparlas	calaspargase pegol	l-asparaginase	*E. coli*	enzyme	5	31–39	leukemia	2018
Palynziq	pegvaliase	phenylalanine lyase	cyanobacteria	enzyme	20	9	phenylketonuria	2018
Esperoct	glycoPEG-antihemophilic factor	factor VIII	human	protein	40	1	hemophilia A	2019
Ziextenzo	pegfilgrastim	G-CSF	human	cytokine	20	1	neutropenia	2019
Udenyca	pegfilgrastim	G-CSF	human	cytokine	20	1	neutropenia	2019

**10:** Comparison of the Prevalence of Anti-PEG Antibodies among Different Studies^197^

**year**	**sample population**	**sample number**	**females/males**	**anti-PEG antibody positive**	**anti-PEG IgM positive**	**anti-PEG IgG positive**	**both IgG and IgM positive**	**assay method and ref**
1984	naïve donors	453	NR	0.2%	NR	NR	NR	hemagglutination^198^
1984	naïve allergy patients	92	NR	3.3%	NR	NR	NR	hemagglutination^198^
2004	naïve donors	250	NR	25%	14%	18%	NR	hemagglutination^199^
2007	gout patients	24	4/20	NR	NR	8.3%	NR	direct ELISA against 10-kDa mPEG-glycine^200^
2011	naïve donors	350	NR	4.3%	NR	NR	NR	bridging assay using hapten-PEG_4000_ ^201^
2014	naïve severe gout patients	30	8/22	19%	NR	NR	NR	direct ELISA against 10-kDa mPEG-glycine + competition ELISA^202^
2015	naïve acute coronary syndrome patients	354	NR	36%	NR	NR	NR	direct ELISA against 10-kDa mPEG-nitrophenyl carbonate + competition ELISA^203^
2015	naïve HBeAg+ subjects	32	NR	6.3%	NR	NR	NR	bridge assay using PEG-IFN or direct ELISA^204^
2016	naïve donors	377	151/226	36.8%^a^	31%^a^	8.5%^a^	2.7%^a^	direct ELISA against DSPE-PEG_5000_ + competition ELISA^205^
2016	naïve donors	1310	NR	23.5%	13.6%	13.5%	NR	direct ELISA against branched PEG_2_0000-HSA^206^
2020	acute lymphocytic leukemia pediatric patients	673	272/401	29.1%	NR	13.9%	NR	flow cytometry assay of beads coated with PEG_5000_ ^207^
2016 and 2021	naïve donors	2404	1209/1195	43.1%^b^	26.4%^b^	25.0%^b^	8.3%^b^	direct ELISA against 10-kDa NH_2_–PEG-NH_2_ + competition ELISA 21 and this report^208^

a Cut-off value of 0.1 μg mL^−1^ for both IgG and IgM.

b Cut-off values of 0.2 μg mL^−1^ for IgG and 0.3 μg mL^−1^ for IgM. NR, not reported.

**Table 11:** Clinically Used Pegylated Nonprotein Drugs^197^

**brand name**	**common name**	**component**	**source**	**type**	**PEG (kDa)**	**PEG number**	**disease**	**year approved**
Doxil	pegylated liposomal doxorubicin (PLD)	doxorubicin	lipid	liposome	2	multiple	cancer	1995
Macugen	pegaptanib	anti-VEGF aptamer	nucleotide	nucleotide	40	1 (branched)	macular degeneration	2004
Movantik	naloxegol	antagonist (C34H53NO11)	drug	small molecule	0.3	1	constipation	2014
Onivyde	irinotecan liposome	irinotecan	lipid	liposome	2	multiple	cancer	2015
Onpattro	patisiran	siRNA in lipid NP	nucleotide	nanoparticle	2.5	multiple	amyloidosis	2018
Comirnaty	tozinameran (BNT162b2)	mRNA in lipid NP	nucleotide	nanoparticle	2	multiple	COVID-19	2020
Moderna COVID-19 vaccine	mRNA-1273	mRNA in lipid NP	nucleotide	nanoparticle	2	multiple	COVID-19	2020

Despite their transformative potential, the widespread adoption of nanobiosensors in biomedical applications faces several critical challenges that must be addressed for successful implementation. One such hurdle is **regulatory approval**, which involves a complex, often lengthy process requiring extensive documentation, clinical validation, and adherence to stringent safety and efficacy standards. The lack of universally accepted regulatory frameworks for nanotechnology-based devices further complicates approval pathways, underscoring the need for standardized testing protocols and clear regulatory guidelines.

Another challenge lies in the **integration of nanobiosensors into existing healthcare systems**. For these technologies to be effective in clinical practice, they must seamlessly connect with hospital infrastructures including electronic health records (EHR) and data management systems. Achieving this requires the development of secure, interoperable platforms capable of handling real-time health data, while maintaining patient privacy and compliance with regulations such as HIPAA or GDPR.

**Cost and accessibility** also present significant barriers. While nanobiosensors offer cutting-edge diagnostic and monitoring capabilities, the high costs associated with their development, production, and deployment can limit their availability, especially in low-resource settings. Addressing this issue calls for innovation in scalable manufacturing techniques, the use of affordable and biocompatible materials, and strategic distribution models that can lower overall system costs without compromising performance.

Nonetheless, nanobiosensors are revolutionizing biomedical applications by offering unparalleled sensitivity, specificity, and real-time diagnostic and monitoring capabilities. Their ability to detect diseases at an early stage, tailor treatments to individual patients, and provide continuous health insights puts them at the forefront of personalized and preventive medicine. While biocompatibility, regulatory hurdles, integration challenges, and affordability remain concerns, rapid progress in nanotechnology, sensor miniaturization, and system integration is steadily overcoming these obstacles. As these advancements continue, nanobiosensors are poised to become foundational tools in delivering more effective, efficient, and accessible healthcare worldwide.

### 3.8 Sustainable Design and Development of Nanobiosensors

#### 3.8.1 Eco-friendly Material Selection and Green Synthesis Methods

Nanobiosensors drive the development of eco-friendly materials and sustainable industrial processes.^209–211^ This is because such sensors allow monitoring, control, and manipulation at the nanoscale, which enables the development of materials and their corresponding processes to have the least possible effect on the environment. For instance, an example might be that nanobiosensors are able to detect and measure pollutants at very low concentrations, thus allowing emissions and wastes in manufacturing procedures to be controlled in real-time. This capability is perhaps what would enable industries to comply with environmental regulations and cleaner production techniques. The potentials of being able to derive sensors at a nano range and embed them in materials so that they could track degradation over time, release, and other environmental factors—so, for instance, these materials break down in some nonpolluting ways and do not release harmful substances—are being used in the green chemistry of nanobiosensors to help develop more sustainable chemical processes. They can trace the progress of chemical reactions and provide optimum conditions for running a reaction at maximum yield with minimum production of waste. For example, nanobiosensors in a biocatalytic field have been crucial in tracing enzyme activity^212^ and substrate conversion in real time, which in turn has allowed tight control over the reaction environment and decreased excess usage of the reagents and energy applied. This leads to more efficient resource usage, thus, reducing the general environmental-related footprint of chemical manufacturing. Through the integration of nanobiosensors into numerous production stages, industries can help to realize more sustainable production lines that diminish dependence upon nonrenewable resources and reduce their impact on the environment.

#### 3.8.2 Energy-Efficient Sensor Architecture and Operation

Nanobiosensors increase the energy efficiency of sensor design by exploiting features intrinsic to high sensitivity and specificity at the nanoscale. Such sensors would require less power to drive while sustaining or improving their performance relative to traditional sensors. Miniaturization of sensing elements reduces the energy required for signal transduction^213^ and data processing.^213^ For instance, in wearable health monitors, nanobiosensors can work within a very low power to detect vital signs continuously, thus extending the battery life of these devices to be more convenient for long-term use. Moreover, the possibility of integration into energy-harvesting systems can make nanobiosensors even more energy-efficient. These systems are then able to capture and transform the ambient energy from sources such as light, heat, or mechanical vibrations into electrical power for the sensors to function. Such a methodology not only reduces the need for external power sources but also enables a whole range of applications, including sensor deployment, in locations that make regular maintenance and battery replacement difficult or uneconomical. Using nanotechnology, those sensors are able to be self-sustaining for very prolonged periods of time while providing continuous monitoring with very low energy demands.^214^ This self-sustaining aspect is especially useful for environmental monitoring, smart cities, and industrial IoT applications—applications where large networks of sensors are deployed to efficiently and sustainably gather data.

#### 3.8.3 Comprehensive Life Cycle Assessment for Environmental Impact

Nanobiosensors themselves undergo life cycle assessment for their environmental impacts, from cradle to grave. This detailed process consists of extraction, processing, and manufacturing of raw materials, usage, and end-of-life disposal or recycling. While reviewing this sequence of stages, LCA detects the most key environmental impacts^215,216^ and odds of improvement. For instance, energy-consuming processes and potentially hazardous chemicals may be assessed during the manufacturing phase in a bid to identify greener alternatives or alternative production methods that are more resource efficient. A number of these options have to focus on bringing down the consumption of resources, reducing emissions to the environment, and not simply making nanobiosensors effective in their applications but also sustainable due to their present pattern of production. More so, deployment and operational phase of nanobiosensors form an important part of their LCA as such. Nanobiosensors designed for low power consumption reduce operational energy use; this is important in their extensive applications, such as environmental monitoring^149^ and smart infrastructure.^217^ The lifetime and durability are also tested to ensure reliability during the intended period of use, eliminating frequent changing that ensures limited electronic wastes. The final life stage provides strategies for recycling or safe disposal for mitigating potential environmental hazards during this stage. It may mean sensors designed with recyclable materials or conversely processes for the safe deconstruction and recovery of valuable components. Considering this, LCA can be done in the design and deployment of nanobiosensors to make such developments more sustainable, considering technological innovation together with care for the environment.

## 4 Challenges and Limitations Identified in Design and Application of Nanobiosensors

Nanobiosensors, characterized by their unparalleled sensitivity, molecular specificity, and real-time response capabilities, are rapidly emerging as pivotal tools across diverse domains, from clinical diagnostics and environmental monitoring to precision agriculture and smart infrastructure systems. Despite their immense promise, the path from conceptualization to commercial translation is fraught with intricate technical, engineering, and translational hurdles. These challenges span the entire innovation lifecycle—ranging from nanoscale fabrication and biological functionalization to system integration, data analytics, and market deployment—and must be systematically addressed to fully harness the transformative potential of these platforms. An in-depth exploration of these multifaceted challenges is presented below (Figure 12), beginning with the fundamental performance determinants of sensitivity and specificity.

**Figure 12:**
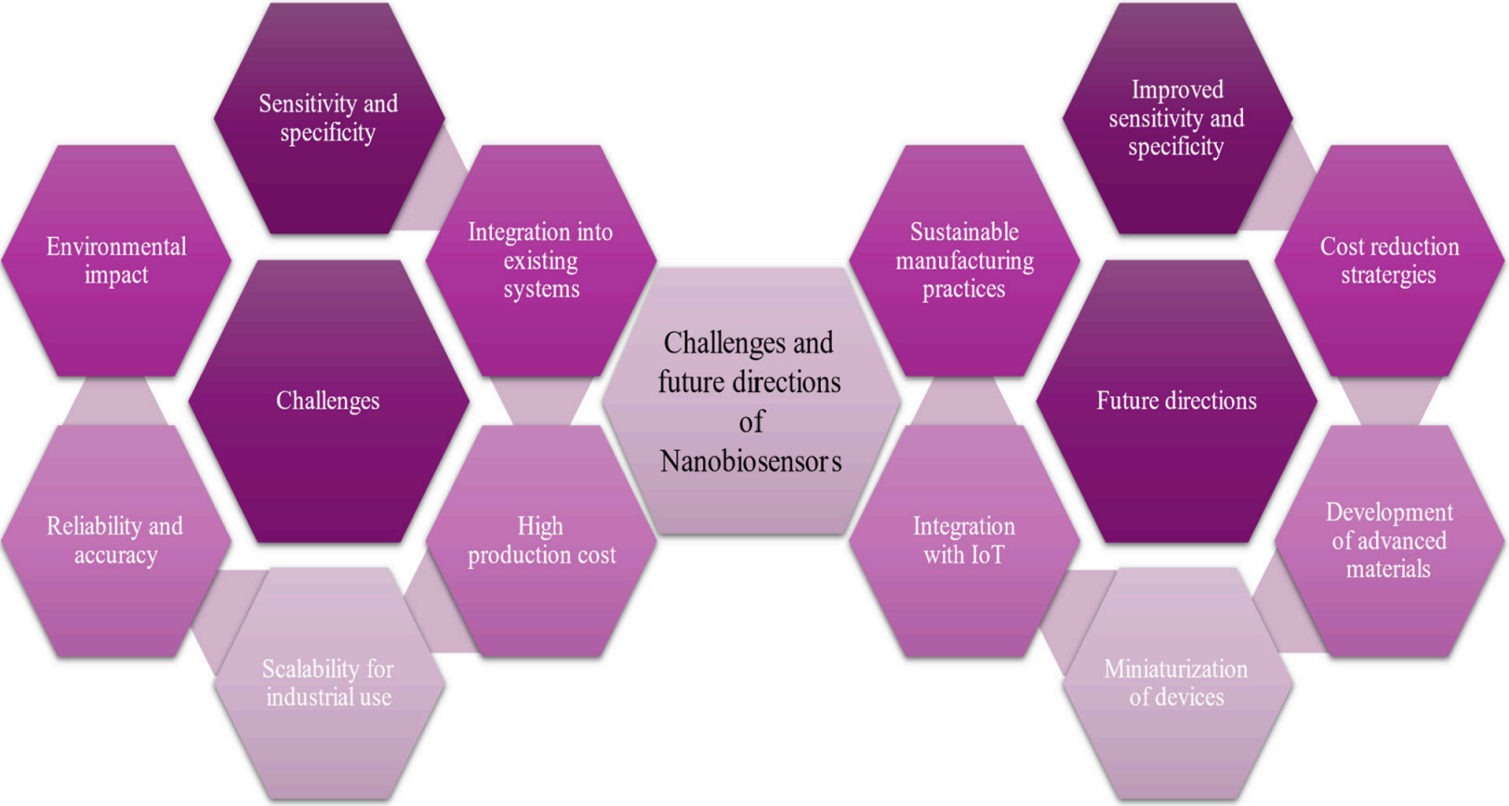
Challenges and future directions of nanobiosensors.

At the core of nanobiosensor performance lies their ability to detect trace quantities of analytes amidst complex biological or environmental matrices. However, while nanoscale sensors inherently exhibit high surface-area-to-volume ratios^218^ that enable ultrasensitive transduction, this very sensitivity can also lead to susceptibility to ambient noise and signal interference, particularly from nonspecific interactions or environmental fluctuations. Such interference can distort output signals and compromise the detection accuracy. To mitigate this, the implementation of advanced noise-filtering algorithms, frequency-domain signal modulation, and on-chip shielding architectures is essential. Moreover, tailoring nanomaterial surface chemistry to reduce nonspecific adsorption and improve target binding fidelity further enhances signal-to-noise ratios under real-world operating conditions.

Maintaining molecular selectivity is equally critical,^219^ especially when distinguishing structurally similar biomolecules within heterogeneous sample matrices. This challenge is amplified at the nanoscale, where minor cross-reactivity can result in significant signal artifacts. Incorporating highly specific biorecognition elements—such as monoclonal antibodies, engineered aptamers, or molecularly imprinted polymers—anchored through covalent linkers or self-assembled monolayers (SAMs) can provide the necessary discriminatory power. Additionally, multiplexed sensor arrays and orthogonal detection mechanisms can improve selectivity while enabling simultaneous multianalyte profiling.

The fabrication and integration of nanobiosensors present another significant bottleneck. Transitioning from lab-scale prototypes to scalable, reproducible, and cost-effective manufacturing processes remains a formidable task due to the intricate alignment of nanoscale materials with biological functionalities. The high-precision requirements of nanolithography,^220^ microfluidic integration, and surface functionalization demand stringent process controls. Emerging manufacturing approaches such as roll-to-roll nanoimprint lithography, aerosol jet printing, and self-assembly based patterning are being explored to address the scalability while preserving device uniformity. Concurrently, hybrid integration strategies that interface nanostructured sensing elements with conventional electronic readout modules must be optimized to ensure minimal signal degradation and seamless electrical transduction. The development of biocompatible interface layers that facilitate efficient electron transfer while preserving the integrity of biomolecular interactions is a critical enabler in this domain.

Ensuring operational durability and environmental stability is essential for applications requiring long-term deployment or in vivo functionality. Nanobiosensors, owing to their size and composition, are inherently susceptible to degradation^221^ under variable temperature, humidity, oxidative stress, or pH conditions. Robust packaging strategies—such as polymeric encapsulation, silica coatings, or metal–organic frameworks (MOFs)—can provide chemical insulation without impeding analyte access or sensor response. For applications involving continuous monitoring, self-healing hydrogels, dynamic surface coatings, or nanocomposite materials that restore sensor integrity postdegradation are being investigated to extend functional lifetimes. To address long-term operational stability, sensors must also incorporate adaptive calibration algorithms and redundancy-based fault detection to maintain accuracy over time and under fluctuating operational loads.

With the increased deployment of sensors across interconnected networks, data management, and computational processing present critical challenges. Nanobiosensors generate high-frequency, high-dimensional data sets that require real-time processing for actionable decision-making. Traditional centralized data systems are often inadequate for such rapid demands. Hence, edge computing architectures, in-sensor data preprocessing, and hardware-accelerated machine learning (ML) inference engines are being developed to enable low-latency analytics. Advanced feature extraction techniques, dimensionality reduction algorithms, and federated learning frameworks can facilitate the efficient handling of streaming data while ensuring user privacy and data integrity.

In parallel, biocompatibility and in vivo safety considerations are paramount, especially for implantable or wearable biosensors. The use of nanomaterials—such as quantum dots, carbon nanotubes, or metal oxides—raises concerns regarding cytotoxicity, oxidative stress, and immune activation. These effects can compromise both sensor performance and biological function. Therefore, the deployment of nontoxic, biodegradable nanomaterials, as well as inert surface passivation layers (e.g., PEGylation or zwitterionic coatings), is crucial to mitigate adverse biological responses. Longitudinal in vivo studies, immunological profiling, and compliance with ISO and FDA biocompatibility testing guidelines are imperative for clinical validation. Equally critical are the regulatory and ethical dimensions associated with nanobiosensor commercialization. The current regulatory landscape lacks harmonized standards for nanomaterial characterization, in vitro and in vivo validation, and risk assessment, resulting in lengthy and uncertain approval timelines. Early stage engagement with regulatory authorities, alignment with ISO, IEC, and FDA guidelines, and participation in standardization consortia can streamline the path to market authorization. Ethical challenges, particularly around data privacy, algorithmic transparency, and the potential misuse of continuous monitoring data, must be addressed through privacy-preserving data architectures, secure communication protocols, and stakeholder-driven ethical frameworks.

Beyond these technical and regulatory challenges, significant commercialization barriers remain that impede the broader deployment of nanobiosensors. High production costs—driven by the complexity of nanofabrication, cleanroom dependency, and bioconjugation protocols—represent a major impediment to market scalability. To address this, innovation in additive manufacturing, low-temperature printing, and modular sensor design is being actively pursued. Techniques such as laser-induced graphene synthesis and solution-processable nanomaterials offer pathways for decentralized, low-cost fabrication.

The regulatory burden, particularly in high-risk applications such as medical diagnostics and environmental surveillance, continues to slow market entry. This is compounded by the lack of standardized performance benchmarks and universally accepted validation protocols for nanoenabled devices. Coordinated efforts to establish international standards, reference materials, and consensus-based evaluation criteria are necessary to lower the regulatory friction and accelerate translation.

Market acceptance and public perception also play decisive roles in determining the success of nanobiosensors. Despite their technical promise, public concerns surrounding the safety of nanomaterials and potential surveillance risks can hinder adoption. Transparent risk communication, community engagement, and the dissemination of empirical safety data are essential to foster public trust. Moreover, awareness gaps among end-users, clinicians, and industry stakeholders often delay adoption. Targeted education campaigns, technology demonstrations, and cross-sector collaborations can help bridge this divide.

System integration and interoperability present additional constraints, particularly when embedding nanobiosensors into legacy systems or IoT frameworks. These sensors must interface seamlessly with existing electronics, data infrastructures, and analytical software. Designing modular, plug-and-play architectures with standardized communication protocols (e.g., BLE, LoRa, and MQTT) can facilitate deployment across diverse environments. Furthermore, establishing cloud-based analytics dashboards and real-time alerting systems can enhance usability and facilitate decision support in clinical and industrial settings.

Finally, achieving economic viability requires demonstrating a compelling value proposition that balances cost with performance and long-term return on investment. While the initial capital expenditure for nanobiosensor systems may be high, downstream benefits—such as reduced healthcare costs through early diagnosis, increased industrial uptime via predictive maintenance, or minimized environmental damage through early pollutant detection—must be clearly quantified. Real-world case studies, health economic modeling, and cost-benefit analyses are essential tools for justifying adoption to potential investors, regulators, and customers.

In conclusion, the development and commercialization of nanobiosensors represent a convergence of cutting-edge science, advanced engineering, and regulatory innovation. Addressing the spectrum of challenges—ranging from nanoscale sensitivity control and biocompatibility to data management and market integration—will require interdisciplinary collaboration, sustained funding, and proactive engagement across academia, industry, and government. As these obstacles are progressively overcome, nanobiosensors are poised to redefine the landscape of diagnostics, environmental monitoring, and real-time sensing, ultimately catalyzing a paradigm shift toward more intelligent, responsive, and personalized systems.

## 5 Future Directions and Opportunities in Nanobiosensors

The future direction and opportunities are presented in subsequent sections.

### 5.1 Emerging Trends and Innovations in Nanobiosensors

Nanobiosensors ride the waves of technological revolution, and emerging trends and innovations increase their potentials and applications. Certainly, one of the key trends is integrating nanobiosensors with the Internet of Things. The convergence of these two concepts—integration into one—can provide real-time monitoring parameters in different environmental settings: from healthcare to industries. In that respect, IoT-enabled nanobiosensors may transmit data wirelessly to any centralized system for continuous monitoring, data analysis, and decision-making. For instance, wearable nanobiosensors in the areas of health care, which are linked with IoT platforms, provide continued monitoring of vital signs and biochemical markers for the early detection of health problems and personalized medical interventions. In environmental monitoring, such sensors track pollutants and changes in the environment in real-time, giving very important information about ecological balance and human health.^164^ Another innovation that is coming up on these lines is highly specific, multifunctional nanobiosensors. Today, how to construct sensors that detect many different analytes with large specificities and sensitivities is achieved by new developments in nanotechnology and molecular biology. Multifunctional sensors will so be particularly useful in complex diagnostic applications^222^ where the simultaneous detection of a variety of biomarkers can give a complete health profile. Further, new developments in the materials science arena—especially the exploitation of graphene and other two-dimensional materials—have allowed improvements in the performance of nanobiosensors through advancements in electrical and mechanical properties. These materials offer high surface area, excellent conductivity, and biocompatibility for sensitive and robust sensor designs. Moreover, artificial intelligence and machine learning algorithms are being integrated with nanobiosensors to boost their ability in data processing and interpretation, which, in turn, provide more accurate and actionable insights. It is these trends and innovations that will keep the drive alive for the evolution of stronger, more versatile, and integral nanobiosensors in different fields.

Nanobiosensors lacking sustainable design often rely on rare or toxic nanomaterials (e.g., Cd-based quantum dots), posing challenges in large-scale manufacturing, environmental disposal, and long-term biocompatibility. Alternatives like carbon-based nanostructures or biodegradable polymers offer greener options with a reduced ecological footprint. However, these substitutes may compromise performance metrics, such as sensitivity or signal stability, necessitating optimization. A critical trade-off emerges between functional efficacy and environmental responsibility, underscoring the urgency for life-cycle assessments and green-by-design frameworks in biosensor development.

### 5.2 Potential for Interdisciplinary Research

Development and application of nanobiosensors are in themselves interdisciplinary,^147^ requiring an input of knowledge from nanotechnology, biology, chemistry, physics, engineering, computer science, and medicine. This collaborative effort is required to deal with the complex issues involved in the design and implementation of such advanced sensors.^147^ For instance, one such result in the arena of health practices could be the development of diagnostic tools that could be highly sensitive and specific by bringing together knowledge from biology that is descriptive of disease markers with the engineering of nanoscale materials and devices. Biologists and chemists might identify relevant biomarkers for diseases, while engineers and physicists could design nanostructures and sensor interfaces for anything that detects these biomarkers with high precision. Furthermore, integration of nanobiosensors with information technology and data science opens up new research frontiers serving interdisciplinary purposes. The data generated by nanobiosensors are huge and complex, requiring advanced data analytics and machine learning techniques for interpretation.^223^ In this regard, algorithms and models for real-time processing of sensor data will be developed with computer scientists and data analysts, in cooperation with engineers and biologists, to enable actions in applications such as personal medicine, environmental monitoring, or process control in industry. Further, the integration of IoT technologies adds expertise in wireless communication and network infrastructure, hence increasing the scope of interdisciplinary research even more. These are so many, very varied fields of collaboration that can exploit the whole potential of nanobiosensors for providing innovative solutions to some of the greatest challenges in health, environment, and industry.

### 5.3 Long-Term Impact on Sustainability

A sustainability contribution to the ecological, economic, and social consideration over the long-term is achieved with the use of nanobiosensors. These sensors environmentally enhance industrial processes that are efficient and less damaging.^147^ They act in time for the monitoring of pollutants and waste products, and for the industry to minimize optimum resource usage, hence reducing their ecological footprints.^149^ An example is the ability of nanobiosensors to monitor soil health^224–231^ and know the state of the crops, enabling precise monitoring and saving on water, fertilizers, and pesticides—an attribute of sustainable farming. This can also ensure early detection and trace environmental pollutants in air and water for remediation attempts or it can ensure protection of the environment and biodiversity.

This also could be a source of economical savings and with a measure for resource efficiency in case the application of the nanobiosensors is widespread. The diagnosis and continuous monitoring offered by the nanobiosensors in health care prevent the advancement of diseases that would have required costly treatments and hospitalization, thereby reducing healthcare cost expenses and improving patient outcomes. In industrial applications, predictive maintenance enabled by nanobiosensors prevents equipment failures and downtime, with anticipated operation savings. These benefits go toward the long-term economics in terms of reduced waste and more efficient use of materials within sectors, therefore contributing to more sustainable economic growth. The benefits of nanobiosensor technologies are in better qualities of life, whereby enhancement of positive health outcomes and living environments are safe. It means options of treatment tailor-made toward personalized medical interventions, raising the effectiveness of the medical intervention. In terms of public health, through real-time detection of pathogens and contaminants, nanobiosensors will prevent outbreaks and ensure a safer availability of foods and water supplies. Further, the data it gathers are both for informing the policy and ensuring the protection of public health and the environment through regulations.

The long-term impact on sustainability will be multifaceted, including environmentally protective, economic efficiency, and social well-being advancements. In view of these facts, nanobiosensors are inherent to the building of a sustainable future with respect to good resource use and the early detection and prevention of any kind of problem. With the support of interdisciplinary research and collaboration, development and deployment of nanobiosensors will continue to make increased contributions toward global sustainability efforts.

## 6 Case Studies and Real-World Applications

### 6.1 Successful Implementations in Civil Engineering

Nanobiosensors have been successfully implemented in civil engineering, particularly in the domain of structural health monitoring. For example, nanobiosensors have been applied to bridges and tunnels by embedding them within the construction material that continuously monitors stress, strain, and the presence of microcracks. Such sensors are capable of sending real-time data regarding a structure’s integrity and hence enable maintenance on time, avoiding a possible grievous failure. For example, San Francisco’s Golden Gate Bridge has fitted nano sensors that monitor continuously to identify the vibration modes.^230^ The study involved the simultaneous measurement of vertical, lateral, longitudinal, and torsional vibrations using strategically deployed accelerometers. A total of 91 modal frequencies were identified on the suspended span: 20 vertical, 18 torsional, 33 lateral, and 20 longitudinal modes, all within the 0.0–1.5 Hz range. The experimental results showed good agreement with 2D and 3D computational models, validating the methodology.^230^ Nanobiosensors are used as internal mix additives in concrete mixtures for real-time monitoring of the curing process, development of cracks, and measurement of the mechanical properties of concrete. To ensure that this technology provided optimal structural performance, it was used in a building which currently is regarded as the world’s tallest structure, Burj Khalifa in Dubai. These implementations show how, actually, nanobiosensors can improve the resilience and reliability of critical infrastructure and hence lead to a safer and more sustainable practice of civil engineering.

### 6.2 Breakthroughs in Biomedical Applications

Nanobiosensors have indeed been significant in the field of biomedicine, particularly in disease diagnosis and monitoring. One pioneering application concerns the early detection of cancer. As such, the capabilities of nanobiosensors for the detection of very low concentrations of cancer biomarkers support an early diagnosis and a personalized treatment plan. For example, a team of scientists from Johns Hopkins University worked out a nanobiosensor that permitted the detection in the blood of DNA mutations related to cancer, offering a noninvasive, highly sensitive diagnostic tool. Another milestone in this respect has been the progress made toward developing glucose monitoring for diabetes management. Nanobiosensors have been developed that provide continuous blood glucose monitoring that provides real-time measurements. This produces a much more accurate and convenient method than the traditional finger prick test. Commercialization companies, such as Dexcom and Abbott, are changing care for people with diabetes by providing better glucose control and lessening the complications that may be a result of it. The discoveries hence vindicate nanobiosensors as having the potential to revolutionize healthcare in due time by coming up with extraordinarily valuable accurate solutions and highly precise diagnosis and monitoring solutions in real-time.

### 6.3 Cross-Sectoral Innovations

Nanobiosensors have enabled cross-sectoral innovations that really underscore their versatility and wide applicability. In environmental monitoring, for example, nanobiosensors are used to monitor air and water pollutants, providing real-time data that is valuable in taking care of environmental protection. A variety of nanobiosensors have been developed that can detect heavy metals and organic pollutants in water sources to facilitate their timely remediation for safe drinking water. The sensors will be installed in cities around the world to track environmental quality and protect public health. Nanobiosensors have applications in the food and agriculture sectors, proving to be important for the safety of the former area and the optimization of practices within the latter. They can detect pathogens and contaminants in food products and prevent foodborne illnesses. Nanobiosensors are further applied in precision agriculture with respect to controlling soil health and crop conditions, whereby farmers can optimize irrigation, fertilization, and pest control to enhance yields while at the same time reducing the environmental impact associated with farming practice. Those very same sectoral innovations underpin the all-rounded contribution that nanobiosensors can make toward improved public health, better environmental protection, higher agricultural efficiency, and safer food. Facilitating real-time monitoring and providing precise detection, nanobiosensors move multiple industries toward a more sustainable and healthy future.

## 7 Electrical Single Molecule Biosensors

Electrical single molecule biosensors are some of the most important types of nanobiosensors. Few researchers like Lv et al., Arachchillage et al., and Williams et al.^231–233^ have conducted elaborate studies on this aspect. This section presents an overview of these studies. Single-molecule sensors, characterized by ultralow detection limits and the ability to resolve stochastic molecular events and heterogeneity, have catalyzed progress in chemical, physical, and biological sciences. Platforms such as nanopores, droplet-based microfluidics, and single-molecule fluorescence microscopy have enabled high-speed, high-throughput molecular interrogation. Among various techniques, molecular counting stands out as the most precise approach for quantifying single-entity behavior. Scanning tunneling microscopy-break junction (STM-BJ) originally developed to investigate quantum electron transport by repeatedly forming metal–molecule–metal junctions—has emerged as a powerful platform for real-time, label-free, and nanoscale-resolved biosensing The tunneling current through such junctions is exquisitely sensitive to the molecular energy landscape, anchoring group–electrode binding geometry, conformational fluctuations, and external stimuli such as electric fields or solvent polarity.

Unlike conventional optical methods such as fluorescence,^234^ surface-enhanced Raman spectroscopy (SERS),^235–238^ and nanopore blockade techniques,^239,240^ STM-BJ sensors benefit from minimal sample requirements, environmental versatility (vacuum, ambient, or solution), and the ability to track interfacial molecular dynamics in real space. Moreover, STM-BJ can resolve conductance quantization, tunneling decay constants, and dynamic bond formation and breakage at femtoampere current resolution. These features enable electronic fingerprinting of target molecules and the construction of quantitative calibration curves based on tunneling conductance histograms. This review presents key STM-BJ-based advancements in sensing ions, environmental pH, and genetic materials, with representative sensor designs summarized in Table 12. We discuss signal transduction mechanisms including molecular orbital alignment, rectification ratios, and thermoelectric signatures that offer multiplexed sensing potential. Strategies such as chemical derivatization, linker engineering, and statistical fitting models enhance specificity and reproducibility in single-event recognition. Despite current limitations to lab-scale demonstrations, the technique holds promise for integration into solid-state platforms for next-generation ultrasensitive biosensors.

**Table 12:** Sensor Types and Their Detection Principles^231^

**Sensors**	**Single-Molecules**	**Detection Target**	**Principle**	**Detection**	**ref.**
pH detection	Dye molecules	pH = 5.5 or 13.6	Change the hybridization of center C atom	Qualitative	^241^
	Cysteine peptides	pH = 6.9 or 2	Protonation/deprotonation of the amine and carboxyl groups	Qualitative	^242^
	Cucurbit[7]uril	pH = 1, 4, 7, 9	Interaction of proton and carbonyl	Qualitative	^243^
	Pyridine derivatives	No details	Protonation or deprotonation of N atom	Qualitative	^244^
	Imidazole	pH = 3, 7, 9, 12	Protonation or deprotonation of N atoms	Qualitative	^245^
	4,4′-vinylenedipyridine	pH = 2.35, 2.57, 2.85, 3.01, 3.26, 3.53	Protonation or deprotonation of N atoms	Qualitative	^246^
	4-(methylthio)benzoic acid	pH = 0 ∼ 5	Protonation or deprotonation of carboxylic acid	Quantitative	^247^
Ion detection	OPE molecule with 15-crown-5 ether or 18-crown-6	metal ions (Li+, Na+, K+, or Rb+)	Host–guest interactions	Qualitative	^248^
	Dithienoborepin	fluoride ion	Lewis acid–base interactions of boron–fluoride	Qualitative	^249^
	3,3′/5,5′-tetramethylbenzidine	Ag[I] (0.2 to 100 μM)	Redox reaction	Quantitative LOD = ∼34 aM	^250^
Genetic materials detection	DNA base pairs	hydrogen-bonding	DNA base pairs for detecting hydrogen-bonding	Qualitative	^251^
	4-mercaptobenzamide	DNA oligomers	Interaction of amino and carbonyl	Qualitative	^252^
	mRNA from *Escherichia coli*	mRNA (from μM to aM)	Complementary base pairing	Quantitative LOD = ∼20 aM	^253^

## 8 Green Nanobiosensing Platforms for Monitoring Biohazards and Toxic Chemical Agents

This section presents overview of studies presented in previous research works.^178^ Scientists have demonstrated significant interest in the development of nanomaterial-based biosensors for detecting environmental pollutants, including heavy metals, pesticides, and pathogens.^254^ Nanomaterials, owing to their high surface-area-to-volume ratio and enhanced catalytic, thermal, and mechanical properties, serve as ideal recognition elements in biosensor construction. These nanomaterial-integrated biosensors exhibit high sensitivity, selectivity, rapid response, and operational stability, allowing for the detection of hazardous substances at extremely low limits of detection. Various nanomaterials—such as quantum dots, metallic nanoparticles, carbon-based nanostructures, and nanoporous metal oxides—have been utilized for environmental pollutant sensing. For instance, Zhou et al. emphasized the efficiency of nanoporous metal/metal oxides in developing gas sensors to monitor pollutants from agricultural and medical activities.^255^ Su et al. further demonstrated that nanoenabled biosensors could efficiently identify organic pollutants in food, water, and agricultural samples,^256^ while Taurozzi and Tarabara reported that integrating silver nanoparticles into sensors significantly enhanced water quality monitoring capabilities.^257^

Among various nanostructures, carbon nanotubes (CNTs) have been widely employed in electrochemical biosensors due to their exceptional surface area, stability, and high sensitivity.^258–260^ CNT-based sensors effectively detect toxic gases like NO_2_, NH_3_, and O_3_, where gas interaction induces a measurable change in the electrical resistance of CNTs through charge transfer or physisorption.^261,262^ Furthermore, these nanomaterial-enabled biosensors extend beyond chemical detection to biological applications; Cesewski and Johnson reported their successful use in detecting environmental pathogens, including toxin-producing algae and sulfur-reducing bacteria. In a related advancement,^263^ Jia et al. developed a sophisticated biosensor by immobilizing whole-cell bioreporters on magnetic nanoparticles (MNPs). This sensor demonstrated high reproducibility in assessing soil toxicity across varying environmental parameters such as pH, salinity, and temperature.^264^ Their field study at a coal cinder site revealed an inverse correlation between soil toxicity and distance from the pollution source, underlining the utility of nanomaterial-integrated biosensors in ecological risk assessment (Table 13).

**Table 13:** Comparison of Various Parameters of Distinct Biosensors for Environmental Pollutants^178^

**Type of biosensor**	**Enzymes/Nanomaterials/Living cells used**	**Electrode/Substrate used for immobilization**	**Target Pollutants**	**Limit of detection (LOD)**	**Stability**	**Working range**	**Application of Biosensor**	**Reference**
Antibiotic detecting biosensors	Amperometric	Pt–Au nanowire array Au(μ-cysteine)-Pt (penicillinase) nanowire array electrode	Penicillin and tetracycline	-	-	-	-	^265^
Pesticides detecting biosensors	Amperometric	Graphene oxide nanosheets Glassy carbon electrode	Hydrazine (HDZ), ascorbic acid (AA) and hydrogen peroxide (H_2_O_2_)	1.04, 0.5, and 4 μM	-	5–100 μM	Investigation of hazardous components in the environment	^266^
	Optical	Whole-cell *Escherichia coli*	3-phenoxybenzoic acid	3 ng/mL	90 days	1.2–1200 ng/mL	Analytical tool to monitor exposure to pyrethroids in environment	^267^
	Optical	*Chlamydomonas reinhardtii* Paper	Nanoencapsulated atrazine	4 pM	21	0.5–200 nM	Help in smart agriculture on site	^268^
	Surface plasmon resonance (Piezoelectric)	Oriented immobilization of immunoglobulin on sensor chip CM5 sensor chip	Chlorpyrifos	0.056 ng/mL	210 cycles	0.25–50.0 ng/mL	Potent device for fast, sensitive, and efficient detection of Chlorpyrifos with wide-ranging uses in The field of environmental monitoring and food safety	^269^
Heavy metallic ions detecting biosensors	Amperometric	DNA-based specific aptamer probes labeled with ferrocene (or methylene blue) and thiol groups at 5′ ad 3′ termini Screen-printed gold	Hg^2+^ and Pb^2+^ ions	0.1 ng/mL	-	0.1–1000 ng/mL	Easy, and inexpensive apta-sensors for fast analysis of heavy metals in water samples	^270^
	Amperometric	Horseradish peroxidase-catalyzed noradrenalin and glucose oxidase enzymes Platinum	Cr^3+^ and Cr^6+^	-	-	-	Investigation of Cr^3+^ and Cr^6+^ ions in real samples	^271^
	Optical	Recombinant plasmids comprised of merR gene	Hg^2+^	1.0 ppb	-	1–104 ppb	Determination of Hg^2+^ ions in the water samples	^272^
	Fluorescence	Aptamer sequence Magnetic beads	arsenic(III) (As^3+^)	2.0 pM	-	0–10 μM	Analysis of As^3+^ in real water samples	^273^
Enzymatic biosensors	Voltammetric	Nitrogen-doped Ordered Mesoporous Carbon	Acetylcholinesterase	0.02 nM	-	3–24 nM	Effective device for quick and sensitive investigation of organophosphorus pesticides in agri-products	^274^

## 9 Conclusions

Nanobiosensors have been able to prove their potential for transformation across various diversified fields, revolutionizing traditional practices and offering a host of new capabilities. For example, nanobiosensors have been used in the structural health monitoring systems of vital civil infrastructure such as bridges, tunnels, and smart cement concrete. They not only supply continual real-time data related to a given structure’s integrity but also pinpoint early stages of stress, strain, and microcracks to make sure that a catastrophic failure does not take place and extends its life cycle. The biomedical applications of nanobiosensors have also seen some very exciting breakthroughs related to disease diagnosis and monitoring. They are capable of detecting diseases such as cancer through the identification of specific biomarkers even at very low concentrations, and continuous glucose monitoring systems have revolutionized diabetes care by replacing periodic blood testing with continuous feedback. Nanobiosensors have played a big role in environmental monitoring, detecting pollutants in air and water to improve timely remediation and save ecosystems. Nanobiosensors are applied in agriculture to food safety via pathogen and contaminant detection and for the optimization of farming practices concerning the health of soils and conditions of crops, which helps in improving yields but reduces environmental impacts. There is, therefore, such versatility and high sensitivity to nanobiosensors that make them very conducive to cross-sectoral innovation, touting applicability in the broad spectrum and ability to drive innovation in public health, environmental sustainability, and industrial efficiency.

### 9.1 Implications for Future Research and Development

The applications of nanobiosensors in these wide-ranging fields open up some key future avenues in research and development. Most important will be the development of large-scale, low-cost processes for the manufacture of nanobiosensors. Currently, nanobiosensors are difficult and expensive to make—a process that keeps them from being widely adopted. New fabrication processes, such as roll-to-roll printing and nanoimprint lithography, might cut costs drastically. Consideration must also be given to standard protocols and procedures to facilitate regulatory approval. This would accelerate acceptance and deployment in critical areas of society including health and environmental monitoring. Another key area is better integration of nanobiosensors with other unfolding technologies, most notably IoT, AI, and related advanced data analytics platforms. Such integrated systems would build real-time monitoring capabilities and data processing for decision-making and make the various systems more responsive and efficient. In this regard, research in biocompatible materials and long-term safety is thus very much required in the area of nanobiosensors, especially for in vivo applications where the sensors can come into direct contact with biological systems. Further research into the life cycle assessment related to nanobiosensors will enhance understanding of their impacts on the environment and the need to minimize the same, while assuring sustainable production and disposal methods. In that respect, the development of nanobiosensors would require interdisciplinary collaboration among nanotechnologists, biologists, engineers, and data scientists.

### 9.2 Synthesis and Outlook: Integrating Sustainability with Technological Innovation

Nanobiosensors have huge potential to enhance sustainability and become a force to drive technological innovation in the modern world. Nanobiosensors present an accurate, real-time monitoring and detection solution that brings enhanced efficiency, safety, and care for the environment in various industries. They improve the resiliency or strength and durability of infrastructure through the early detection of structural issues in civil engineering. In healthcare, they aid early diagnosis and facilitate personalized treatment; hence, significantly improving patient outcomes. With the timely detection and remediation of pollutants, environmental monitoring safeguards ecosystems and public health. Nanobiosensors optimize the use of resources and enhance food safety in agriculture by providing minute information about soil and crop conditions. Integration with IoT and AI further expands their effect, hence, allowing for the development of smarter, more efficient systems. Challenges to be addressed with regard to cost, regulatory approvals, integration, biocompatibility, and environmental impact need to be overcome before nanobiosensors can reach their full potential. Interdisciplinarity and innovation will unleash the complete potential of nanobiosensors; with such driving forces behind sustainable development with positive impact on society and the environment. Also, further development and acceptance of nanobiosensors will be very critical in building a sustainable future wherein technology and nature go hand in hand to promote human well-being and environmental health.

The integration of nanobiosensors across biomedical and civil engineering domains demands a comprehensive evaluation of their environmental sustainability, encompassing materials sourcing, fabrication methods, lifecycle impacts, and end-of-life strategies. Many current nanobiosensor platforms rely on rare-earth elements, heavy metals (e.g., Cd, Pb, Hg), or synthetic polymers with low biodegradability, raising serious concerns about environmental persistence and toxicity. For instance, commonly used quantum dot-based sensors involve cadmium telluride or indium phosphide, both of which pose challenges for biocompatibility and ecotoxicity if improperly disposed of or released in trace quantities. Furthermore, solvent-intensive fabrication methods (e.g., wet-chemical synthesis, spin-coating, lithography) often generate hazardous byproducts, which are rarely recovered or treated in lab-scale and field-deployed systems. A rigorous life cycle assessment (LCA), including upstream and downstream emissions, energy use, and waste footprint, remains absent from most current biosensor development pipelines, impeding the transition toward environmentally responsible sensor networks.
